# A databank for intracellular electrophysiological mapping of the adult somatosensory cortex

**DOI:** 10.1093/gigascience/giy147

**Published:** 2018-12-06

**Authors:** Angelica da Silva Lantyer, Niccolò Calcini, Ate Bijlsma, Koen Kole, Melanie Emmelkamp, Manon Peeters, Wim J J Scheenen, Fleur Zeldenrust, Tansu Celikel

**Affiliations:** Department of Neurophysiology, Donders Institute for Brain, Cognition and Behaviour, Radboud University, Heyedaalseweg 135, 6525 HJ, Nijmegen - the Netherlands

**Keywords:** whole-cell intracellular recordings, somatic patch-clamp, current-clamp, voltage-clamp, acute brain slices, adult brain, barrel cortex, frozen noise, big data

## Abstract

**Background:**

Neurons in the supragranular layers of the somatosensory cortex integrate sensory (bottom-up) and cognitive/perceptual (top-down) information as they orchestrate communication across cortical columns. It has been inferred, based on intracellular recordings from juvenile animals, that supragranular neurons are electrically mature by the fourth postnatal week. However, the dynamics of the neuronal integration in adulthood is largely unknown. Electrophysiological characterization of the active properties of these neurons throughout adulthood will help to address the biophysical and computational principles of the neuronal integration.

**Findings:**

Here, we provide a database of whole-cell intracellular recordings from 315 neurons located in the supragranular layers (L2/3) of the primary somatosensory cortex in adult mice (9–45 weeks old) from both sexes (females, N = 195; males, N = 120). Data include 361 somatic current-clamp (CC) and 476 voltage-clamp (VC) experiments, recorded using a step-and-hold protocol (CC, N = 257; VC, N = 46), frozen noise injections (CC, N = 104) and triangular voltage sweeps (VC, 10 (N = 132), 50 (N = 146) and 100 ms (N = 152)), from regular spiking (N = 169) and fast-spiking neurons (N = 66).

**Conclusions:**

The data can be used to systematically study the properties of somatic integration and the principles of action potential generation across sexes and across electrically characterized neuronal classes in adulthood. Understanding the principles of the somatic transformation of postsynaptic potentials into action potentials will shed light onto the computational principles of intracellular information transfer in single neurons and information processing in neuronal networks, helping to recreate neuronal functions in artificial systems.

## Data Description

The primary somatosensory cortex encodes time-varying but spatially well-defined haptic information [1] from the mechanoreceptors in the skin, thereby creating a topographical neuronal representation of the tactile world [2, 3]. Rodents, e.g., locate tactile targets in their immediate environment by integrating information across these (whisker) representations in the barrel cortex [4], where neurons in each cortical column preferably respond to a single whisker on the contralateral snout [5]. The supragranular layers (cortical layers 2/3, L2/3) of the barrel cortex are the first cortical network that integrates the sensory information across neighboring cortical columns, whiskers, and whisk cycles [6–10]. This representation of the whisker contacts undergoes experience-dependent changes [11–14] and is altered in animal models of neurodevelopmental disorders [15–17]. Adaptive changes in the synaptic and modulatory drive could powerfully regulate the transformation of postsynaptic responses into action potentials, ultimately controlling how sensory information is transferred between cortical columns and cortical regions [18].

Understanding the principles of neuronal information transfer in the supragranular layers will require a systematic analysis of the integrative properties of these cortical neurons. Thus far, however, slice experiments primarily focused on juvenile animals as it is widely considered that the neurons mature anatomically and electrophysiologically by the fourth postnatal week [16, 19–23]. Here, we provide a database of 837 experiments collected from 315 adult supragranular neurons that will help to address the principles of information processing by cortical neurons throughout the adulthood of mice. The database consists of whole-cell intracellular recordings in voltage-clamp (VC) and current-clamp (CC) configurations. While CC somatic measurements bring insight into the properties related to action potential initiation, timing, rate, and pattern, VC recordings provide information on the voltage-gated ion-channel dynamics. The database is best utilized to address the principles of information transfer in individual neurons (see, e.g., [18, 24]) and for the electrical characterization of adult cortical sensory neurons. It will serve synaptic, systems, computational, and theoretical neuroscientists in search of the principles of information processing, transfer, and recovery in neuronal networks. The database is expected to create synergy with the recently completed transcriptome [25, 26] and proteome [27, 28] of the supragranular layers of the barrel cortex, the computational models of the molecular changes that contribute to the maturation of synaptic communication in the same cortical region (e.g., [29]), the computational models of synaptic integration and action potential generation in the supragranular layers of the barrel cortex [18], and the high-resolution mapping of sensory representations using intrinsic signals in single trial resolution (e.g., [30]) resulting in a multi-scale analysis of the cortical organization, from molecules of chemical communication to network representations.

## Methods

Experiments that involve animals were conducted in accordance with the European Directive 2010/63/EU, national regulations in the Netherlands, and international guidelines on animal care and use of animals. Pvalbtm1(cre)Arbr (RRID:MGI:5315557) or Ssttm2.1(cre)Zjh/J mice (RRID:IMSR_JAX:013044) from either sex (N = 75 females, N = 45 males, aged 9–45 weeks) from the local breeding colonies were used.

**Figure 1:**
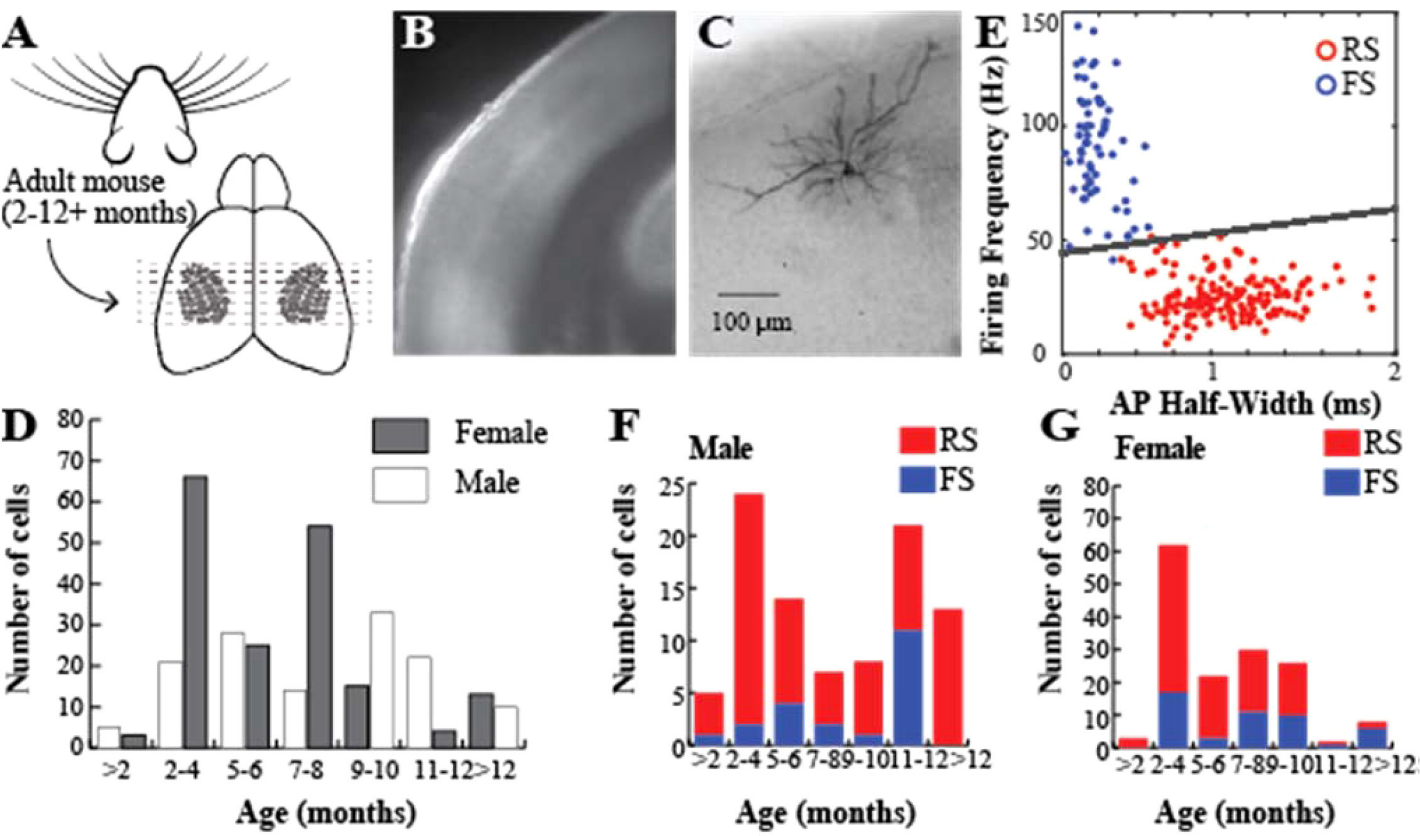
Acute slice preparation. **(A)** Coronal slices (300 micrometers in thickness) were prepared for *ex vivo* recording from the L2/3 neurons in the mouse primary somatosensory cortex, barrel cortex subregion. **(B)** A low magnification view of the slice in 4x. **(C)** A representative neuron, intracellularly filled with biocytin and visualized with DAB staining (VECTASTAIN Elite ABC Kit, RRID:AB_2336827) according to the manufacturer's guidelines. **(D)** Distribution of the 326 neurons in this database across males (39.9%) and females (60.1%) as well as the ages of the animals. **(E)** Classification of the neurons presumed fast-spiking (FS) and regular spiking (RS) populations based on firing frequency and action potential half-width (see Methods section for details). **(F-G)** The distribution of cells across cell type and ages.

The mice were anesthetized with Isoflurane (1.5 mL/mouse) before the tissue was extracted and coronal slices of the primary somatosensory cortex, barrel subfield region, were prepared (Fig. 1). The procedures were as described elsewhere [11, 12, 14, 16, 31] with the exception that animals were intracardially perfused with ice-cold dissection solution containing (in mM) 108 choline chloride, 3 KCl, 26 NaHCO_3_, 1.25 NaH_2_PO_4_·H_2_O, 25 glucose·H_2_O, 1 CaCl_2_·2H_2_O, 6 MgSO_4_·7H_2_O, and 3 sodium pyruvate after animals were deeply anesthetized, as assessed by pinch withdrawal reflex and heart and breathing rate. The brain was removed after decapitation and sliced coronally (300 micrometers in thickness) in the same ice-cold perfusion medium. The slices were then transferred to a chamber containing artificial cerebrospinal fluid (aCSF) (in mM): 120 NaCl, 3.5 KCl, 10 glucose·H_2_O, 2.5 CaCl_2_·2H_2_O, 1.3 MgSO_4_·7H_2_O, 25 NaHCO_3_, and 1.25 NaH_2_PO_4_·H_2_O and aerated with 95% O_2/_5% CO_2_ at 37°C. After 30 minutes, the slices were transferred to room temperature before whole-cell electrophysiological recordings started.

### Whole-cell recordings

**Figure 2:**
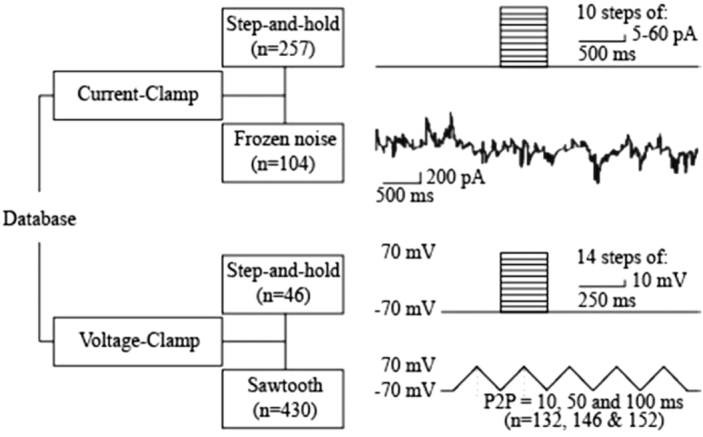
Experimental protocols and the hierarchical organization of the database. The data are available online at [36]·. The database contains two subfolders, current-clamp and voltage-clamp, each of which has additional subfolders based on the stimulus protocols utilized in this study. Each dataset is provided in a .mat format and includes both voltage and current channels unless otherwise described. The stimulus delivered to the cells as well as the cell's response can be quantified from these variables.

Slices were continuously oxygenated and perfused with aCSF during recordings. The barrel cortex was localized, and cells of interest in the supragranular layers were patched under 40x magnification in room temperature using HEKA EPC 9 and EPC10 amplifiers in combination with the Patch Master v2 × 90.2 data acquisition software. The data band-pass filtered 0.1–3000 Hz. The AC mains (hum) noise (max peak-to-peak amplitude 0.2 mV) that exists in a subset (∼4%) of the recordings was not filtered. Patch-clamp electrodes were pulled from glass capillaries (1.00 mm [external diameter], 0.50 mm [internal diameter], 75 mm [length], GC100FS-7.5, Harvard Apparatus) with a P-2000 puller (Sutter Instrument, USA) and used if their initial resistance was between 5 and 10 MOhm. They were filled with intracellular solution containing (in mM) 130 K-Gluconate, 5 KCl, 1.5 MgCl_2_·6H_2_O, 0.4 Na3GTP, 4 Na2ATP, 10 HEPES, 10 Na-phosphocreatine, and 0.6 EGTA, and the pH was set at 7.22 with KOH. CC and VC recordings were performed as described elsewhere [32, 33] and included four stimulus protocols (Fig. 2).

### Current-clamp protocol

After establishing the CC configuration, the resting membrane potential was set to −70 mV by direct somatic current injections, as required. The step-and-hold stimulation protocol included 10 steps of 500 ms long depolarization pulses (step size: 5, 10, 20, 40, or 60 pA) with an inter-sweep interval of 6.5 s. The stimulus train was repeated 1–3 times with a 20 s interval. The drift, if any, in resting membrane potential during the recording was not corrected for. However, any neuron whose resting membrane potential varied more than 7 mV was not included in the database. The frozen-noise (FN) stimulation protocol involved somatic injection of the current that is the output of an artificial neural network of 1,000 neurons, each firing Poisson spike trains in response to a “hidden state” (see [34] for details and [35] on how to generate the FN input and analyze the data).

### Voltage-clamp protocol

The VC stimulation protocols included step-and-hold and sawtooth (triangular) pulse injections (Fig. 2). In both protocols, the membrane potential was clamped at −70 mV prior to somatic depolarization. In the step-and-hold protocols, 14 incremental steps of depolarizing pulses (10 mV/each) were delivered for a period of 250 ms with an interval of 20 s. Sawtooth pulses (range: −70 to 70 mV) were delivered at three frequencies (5, 10, and 50 Hz) and consisted of five triangular pulses with peak-to-peak distances of 200, 100, and 20 ms, respectively. Each trial was repeated twice with 20 s interval.

### Data organization

Files in “.mat” (MATLAB) format containing the original traces from each experiment are organized in folders separated by the structure described in Fig. 2. Metadata including the date and number of the experiments, the experimenter's initials, the animal's sex and age, the experimental protocol, the cell type, and the animal number are included in a tabulated format (.xlsx, Microsoft Excel; Supplementary Table S1). The experiments are named as the date_prefix_experiment number_protocol number. All cells recorded from the same animal share the same experimental date.

The CC data (see “Current Clamp” folder) contain two subfolders, Step Protocol and Frozen Noise. Step Protocol data include two channels (voltage and current), each of which includes two columns (timestamp and voltage/current values in volt and amp, respectively) for each repetition. Users can visualize both the current injected to clamp the soma and the observed voltage response. Data from each stimulus condition are saved under a separate variable that starts with “Trace_a_b_c_d” and includes information about (a) the cell and experiment ID, (b) the data type, (c) the number of sweeps in each dataset, and (d) the channels.

The Frozen Noise subfolder contains the voltage trace (i.e., neuronal response to the injected FN), hidden state (activity in the modeled network responds to, see [34] for details), and the injected current trace. In addition, a Matlab “struct” variable named “settings” is provided. Settings provide metadata under the following “fields”: condition, experimenter, baseline (membrane potential value [in mV] at which the cell is kept with the baseline current injection), amplitude_scalind (the scaling factor used to translate the output of the neural network, in pA value), tau (the time constant that defines the average switching speed of the hidden state), mean_firing_rate (of the artificial neurons), sampling_rate (the acquisition rate [in kHz]), duration [in ms], FLAG_convert_to_amphere (a binary value that is 1 if the output was converted into ampere), and cell_type (regular spiking vs fast spiking).

The VC folder includes two subfolders: VC Step (voltage step-and-hold) and VC Sawtooth, the latter containing three subfolders with recordings from experiments with triangular sweeps at three frequencies (5, 10, or 50 Hz). Data in the Voltage Clamp folder is organized similar to data in the Current Clamp folder, and variable naming follows the formatting rules described above.

#### Cell type classification

K-means clustering (cluster count = 2; the number of repetition = 10) was performed to classify neurons into fast-spiking and regular-spiking neurons, using CC step-and-hold recordings. The clustering was based on the maximum firing rate reached during the current step injections and on the mean spike half-width across all stimulus steps during the CC step-and-hold protocol. Please note that the cell classification is solely provided to help the user navigate the data. We do not claim that neurons can be necessarily electrically classified in a binary fashion nor do we claim that commonly utilized clustering approaches are optimal for accurate (albeit broad) classification of excitatory (mostly regular spiking) and inhibitory (predominantly fast-spiking) neurons.

### Re-use potential

The dataset is rich in information regarding current vs voltage dynamics in adult cortical neurons. The independent variables in the database are the sex and age of the animal. While CC experiments provide information about sub- and suprathreshold voltage dynamics, the VC experiments are informative about the ionic conductances that lead to activation or inactivation of neurons.

**Figure 3:**
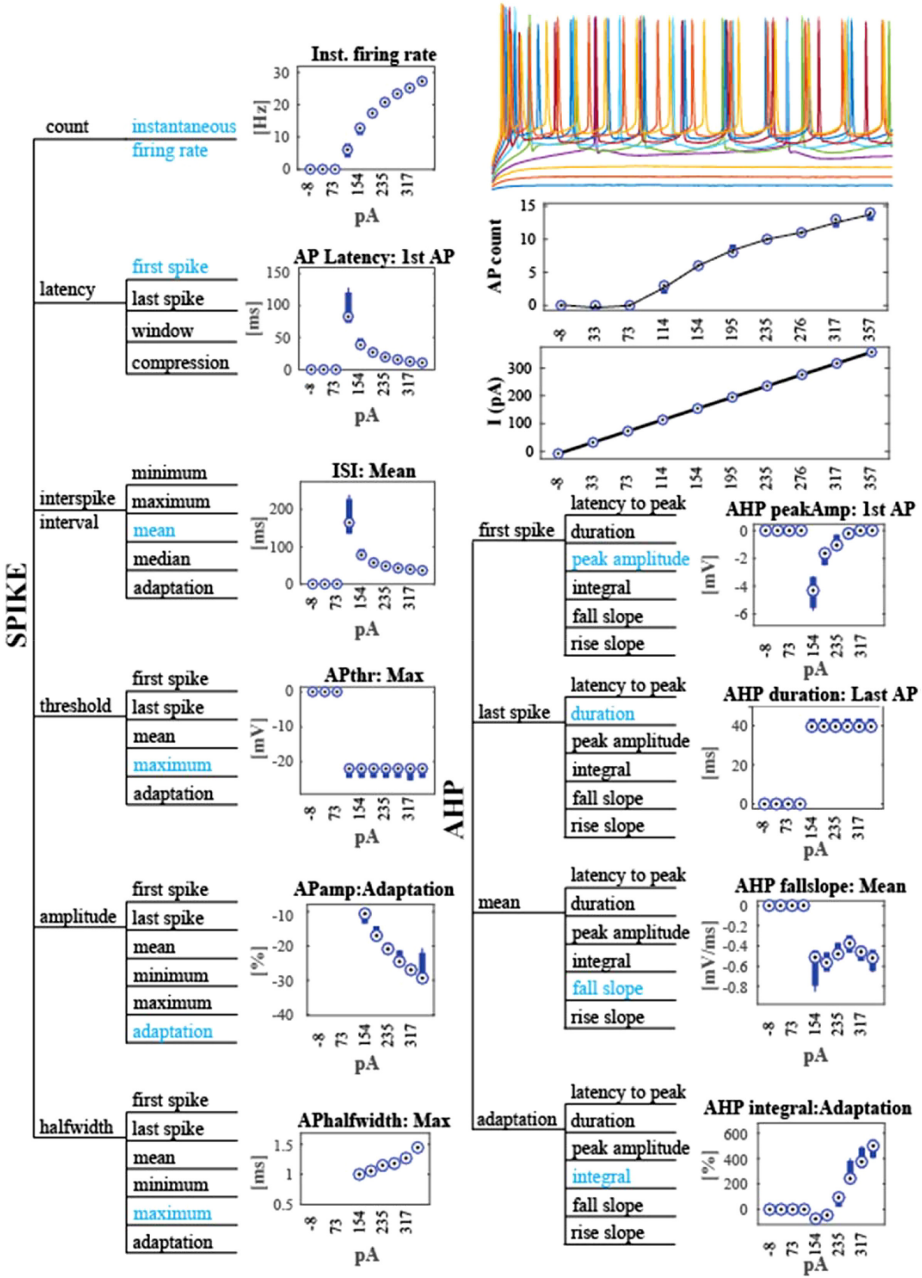
Electrical characterization of the spiking response in CC experiments. The parameter space is shown as a hierarchical tree. Variables shown in blue are used for the data displays. AHP = afterhyperpolarization. “Compression” is a normalized metric that can be calculated as the difference between observations (e.g., spike timing) over the duration of stimulus. In the case of “latency compression,” it is calculated as the temporal difference between the first and last action potential divided by the stimulus duration. “Adaptation” is the relative change in the observed variable, normalized to the first event. For example, in the case of spike amplitude adaptation, it is calculated as (AP_amp_first-AP_amp_last)/AP_amp_first (AP, action potential). All characteristics are measured relative to the stimulus amplitude (the current injected, in pA). Membrane potential traces on the top right are the responses to incremental current injections superimposed on top of each other. The data below the raw traces represent the number of action potentials and the amplitude of the injected current across the 10 step-and-hold stimuli in this experiment (Filename: 170130_AL_133).

In the step-and-hold CC experiments, the voltage responses can be quantified using subthreshold (e.g., amplitude, latency, duration of the postsynaptic potential) and suprathreshold (e.g., interspike interval adaptation, spike count, spike half-width) responses to somatic current injection (Fig. 3). Because multiple stimuli with incrementally increasing current intensities are delivered, cellular responses can be mapped onto stimulation intensities, allowing users to study input/output curves for the parameters of interest.

Action potentials can be studied both in terms of their shape (e.g., waveform, rise and decay slope, amplitude of the positive and negative peaks, the half-width of spike) and temporal response properties (that allow quantification of the rate and timing of action potentials during synaptic activation). Since adaptation to a sustained current injection is commonly used as a criterion to classify neurons, the data provide an inclusive database for the electrical classification of adult neurons, creating synergy with other publicly available databases, e.g., Neurodata Without Borders [37] and the Allen Institute Cell Type database [38]. The data can be used independently or in the context of computational models of neural networks, a broad selection of which can be found in the ModelDB database [39].

**Figure 4:**
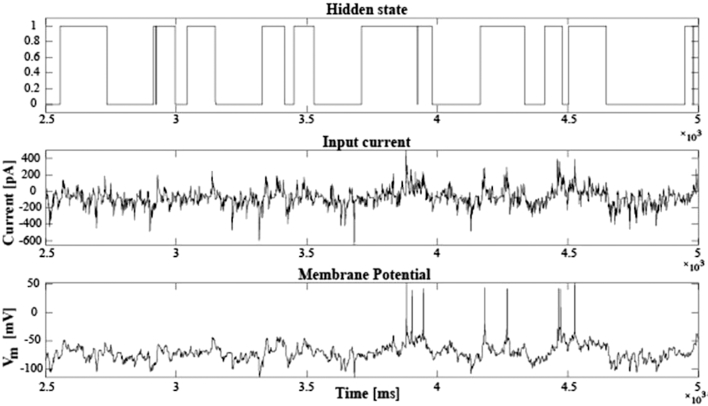
Frozen-noise injection in CC configuration. Representative recording from a single neuron (experiment 171207_NC_146). Top row: Binary representation of the hidden state that forms the input to an artificial neural network with 1,000 point neurons, firing action potentials following an inhomogeneous Poisson process (see [34] for details). Middle row: The synaptic current generated by the artificial network that was injected into the recorded neuron. Bottom row: The membrane potential response of the recorded neuron.

In addition to sustained somatic depolarization, the CC database also includes FN injections, during which a time-varying current was injected into the recorded neuron (Fig. 4). The injected current was generated using an artificial neural network (see [34] for details) of 1,000 neurons, each one firing spike trains from an inhomogeneous Poisson process, responding to a binary hidden state that represents the presence or absence of an external stimulus. The activity of all the neurons in the artificial network is integrated, and the resulting current is corrected for the baseline current required to keep the patched neuron at −70mV. This summed current is injected to the patched soma. A major utility of the FN protocol is that it allows direct quantification of neuronal information transfer [10, 34]. Compared to other metrics of neuronal information transfer [18, 40, 41], this approach enables bias-free quantification of information with a short (3 or 6 minutes) stimulation protocol [34].

In the database, experimental data recorded from our FN protocol include the recorded membrane potential voltage, the hidden state, and the current injected into the neurons (Fig. 4). Thus, the user can perform forward and reverse modeling to predict the neuronal response and to study neuronal dynamics in the adult neocortex.

**Figure 5:**
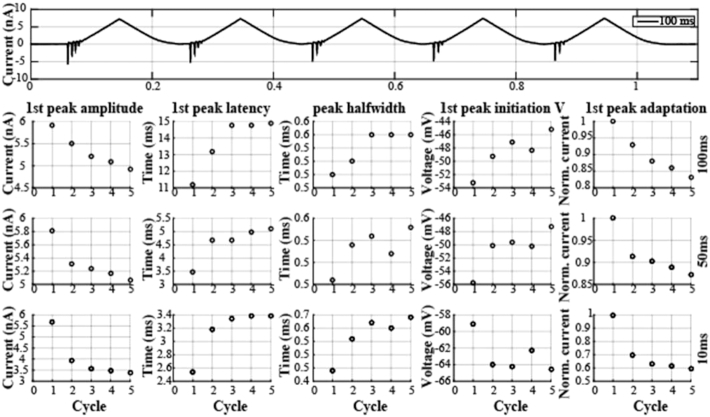
Voltage-clamp sawtooth protocol. Top row: Current trace from a representative experiment (180412_AB_53_ST). Figurines, left to right, are measurements of the first peak amplitude, first peak latency, the half width of the first inward current, membrane potential at which the inward current is initiated, and the adaptation of the first event amplitude across the five (triangle) cycles. Data in the bottom three rows are from three different sawtooth speeds (10/50/100 ms, corresponding to 100/20/10 Hz stimulation). The five points in each figurine are calculated from the first inward current in each (triangle) cycle.

Going beyond the voltage dynamics in the adult neurons, the database also provides insight into the ionic currents that flow through the membrane. With the triangle-shaped VC-Saw protocol (Fig. 5), it is possible to measure the activation threshold of the currents flowing through the membrane by assessing when deviations from the expected sawtooth shape occur. Additionally, it is possible to compute amplitudes and latencies of the events, peaks half-widths, the percentage difference between consecutive events, and the total number of events in each sweep. Other features could also be extracted from the dataset depending on the researchers' interests.

**Figure 6:**
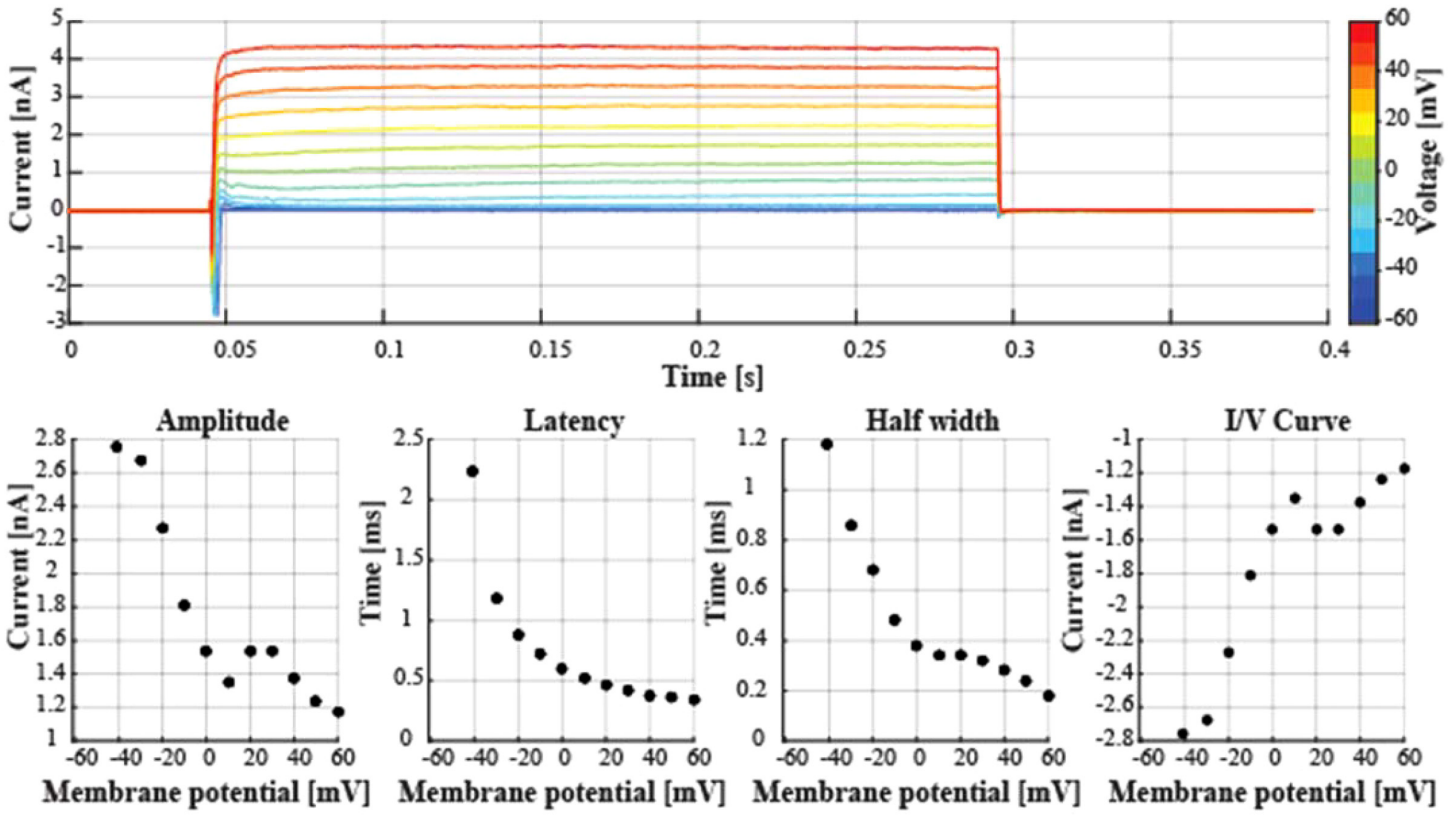
Step-and-hold protocol in voltage-clamp preparation. Top panel shows data from a representative experiment (170915_AB_5_VC). Every other figurine shows one of the analyzed features, including the amplitude of the peak, temporal delay between the stimulus onset and the peak amplitude (i.e., latency), width of the evoked transient measured at half maximum, as well as the current at every holding potential (I/V curve).

The current-voltage relationship was measured with VC steps (Fig. 6), which could be used to produce an current/voltage (I/V) curve. The peak amplitude, latency, and peak half width can be extracted for the inward currents observed during the sustained depolarization of the soma.

## Availability of source code and requirements

Project name: Rapid mutual information calculation using frozen noise injection

Project home page: https://github.com/DepartmentofNeurophysiology/Analysis-tools-for-electrophysiological-somatosensory-cortex-databank[35]

Operating system: Platform independent

Programming language: MATLAB

Other requirements: MATLAB version 2017a or higher.

License: GNU GPL

RRID:SCR_016558

## Availability of supporting data

Snapshots of the database and code, including additional supporting data, are available in the *GigaScience* repository, GigaDB [36].

## Additional file

Lantyer_Supplemental Table_Metadata.xlsx

## Application scenarios

From the recordings available in this database, it is possible to actively quantify the membrane properties of supragranular layer neurons to infer current and voltage dynamics during somatic depolarization.

Network development is based on processes of self-organization that are highly dependent on sensory stimuli and experience [2]. Such plasticity is not limited to early life development. From the VC and CC experiments, it is possible to infer the biophysical properties of layer 2/3 pyramidal neurons of the adult somatosensory cortex under baseline conditions in the absence of altered sensory experience.

The focus on adult neurons brings a new perspective to the study of membrane properties, as data from this mature age are still scarce. The dynamics of the active electrical properties of the membrane can be accessed as a function of different developmental time points and/or sex, and the recorded data can be used as virtual neurons in dynamic-clamp experiments.

In a computational approach, spiking properties described herein could be used for biomimetic modeling of diverse networks, facilitating the study of computational roles of circuit motives. Moreover, applying the principles of information transfer and recovery to the data might help recreate neuronal functions in artificial systems.

## Limitations

Neurons in this dataset originate from regular-spiking and fast-spiking neurons; however, there is no anatomical characterization of the neuron type studied. The database is focused on layer 2/3 of the somatosensory cortex as a model region and does not allow the study of neuronal information processing across different cortical regions in isolation. However, the user might consider comparing data across different regions and species by utilizing the other publicly available databases, e.g., Neurodata Without Borders [37], the Cell Type database [38] of the Allen Institute and the Collaborative Research in Computational Neuroscience data sharing initiative [42].

## Abbreviations

aCSF: artificial cerebrospinal fluid; AP: action potential; CC: current clamp; FN: frozen noise; I/V: current/voltage; L2/3: cortical layer 2/3; VC: voltage clamp

## Competing interests

The authors declare that they have no competing interests.

## Funding

This work was supported by a doctoral fellowship from the National Council for Scientific and Technological Development of Brazil (CNPq) to A.S.L.; grants from the European Commission (Horizon2020, nr. 660328), European Regional Development Fund (MIND, nr. 122035), and the Netherlands Organisation for Scientific Research (NWO-ALW Open Competition, nr. 824.14.022) to T.C.; and by the Netherlands Organisation for Scientific Research (NWO Veni Research Grant, nr. 863.150.25) to F.Z.

## Author contributions

**Table:** 

**Contributor Role**	**Author**
**Conceptualization**	Tansu Celikel (TC)
**Supervision**	Koen Kole (KK), Wim JJ Scheenen (WJJS), Fleur Zeldenrust (FZ), TC
**Project Administration**	TC
**Investigation**	Angelica da Silva Lantyer (ASL), Niccolò Calcini (NC), Ate Bijlsma (AB), KK, Melanie Emmelkamp (ME), Manon Peeters (MP)
**Formal Analysis**	ASL, NC, AB
**Software**	NC, FZ, TC
**Methodology**	FZ, TC
**Validation**	ASL, NC, AB
**Data Curation**	ASL
**Resources**	TC
**Funding Acquisition**	ASL, FZ, TC
**Writing—Original Draft Preparation**	ASL, TC
**Writing—Review & Editing**	ASL, NC, AB, ME, MP, KK, WJJS, FZ, TC
**Visualization**	ASL, NC, AB, FZ, TC

## Supplementary Material

GIGA-D-18-00318_Original_Submission.pdf

GIGA-D-18-00318_Revision_1.pdf

GIGA-D-18-00318_Revision_2.pdf

GIGA-D-18-00318_Revision_3.pdf

Response_to_Reviewer_Comments_Original_Submission.pdf

Response_to_Reviewer_Comments_Revision_1.pdf

Response_to_Reviewer_Comments_Revision_2.pdf

Reviewer_1_Report_(Original_Submission) -- Janos Fuzik 9/7/2018 Reviewed

Reviewer_1_Report_(Revision_1) -- Janos Fuzik 10/29/2018 Reviewed

Reviewer_2_Report_(Original_Submission) -- Hongdian Yang 9/14/2018 Reviewed

Reviewer_3_Original_Submission_(Attachment).pdf

Reviewer_3_Report_(Original_Submission) -- Suhasa Bangalore Kodandaramaiah 10/2/2018 Reviewed

Supplemental File

## References

[1] Azarfar A, Zhang Y, Alishbayli A An open-source high-speed infrared videography database to study the principles of active sensing in freely navigating rodents. GigaScience. 2018, Dec 1;7(12). 10.1093/gigascience/giy134. PMC6283211 30418576

[2] Kole K, Scheenen W, Tiesinga P Cellular diversity of the somatosensory cortical map plasticity. Neurosci Biobehav Rev. 2018;84:100–15. 29183683 10.1016/j.neubiorev.2017.11.015

[3] Diamond ME, Petersen RS, Harris JA Learning through maps: functional significance of topographic organization in primary sensory cortex. J Neurobiol. 1999;41:64–8. 10504193

[4] Celikel T, Sakmann B Sensory integration across space and in time for decision making in the somatosensory system of rodents. Proc Natl Acad Sci U S A. 2007;104:1395–400. 17227858 10.1073/pnas.0610267104 PMC1783091

[5] Van der Loos H, Woolsey TA Somatosensory cortex: structural alterations following early injury to sense organs. Science. 1973;179:395–8. 4682966 10.1126/science.179.4071.395

[6] Voigts J, Herman DH, Celikel T Tactile object localization by anticipatory whisker motion. J Neurophysiol. 2015;113:620–32. 25339711 10.1152/jn.00241.2014

[7] Carvell GE, Simons DJ Task- and subject-related differences in sensorimotor behavior during active touch. Somatosens Mot Res. 1995;12:1–9. 7571939 10.3109/08990229509063138

[8] Voigts J, Sakmann B, Celikel T Unsupervised whisker tracking in unrestrained behaving animals. J Neurophysiol. 2008;100:504–15. 18463190 10.1152/jn.00012.2008

[9] Carvell GE, Simons DJ Effect of whisker geometry on contact force produced by vibrissae moving at different velocities. J Neurophysiol. 2017;118:1637–49. 28659457 10.1152/jn.00046.2017 PMC5577550

[10] Azarfar A, Calcini N, Huang C, Neural coding: a single neuron's perspective. Neurosci Biobehav Rev. 2018;94:238–47. 30227142 10.1016/j.neubiorev.2018.09.007

[11] Allen CB, Celikel T, Feldman DE Long-term depression induced by sensory deprivation during cortical map plasticity in vivo. Nat Neurosci. 2003;6:291–9. 12577061 10.1038/nn1012

[12] Celikel T, Szostak VA, Feldman DE Modulation of spike timing by sensory deprivation during induction of cortical map plasticity. Nat Neurosci. 2004;7:534–41. 15064767 10.1038/nn1222 PMC3082358

[13] Foeller E, Celikel T, Feldman DE Inhibitory sharpening of receptive fields contributes to whisker map plasticity in rat somatosensory cortex. J Neurophysiol. 2005;94:4387–400. 16162832 10.1152/jn.00553.2005 PMC3070316

[14] Clem RL, Celikel T, Barth AL Ongoing in vivo experience triggers synaptic metaplasticity in the neocortex. Science. 2008;319:101–4. 18174444 10.1126/science.1143808

[15] Juczewski K, von Richthofen H, Bagni C, Somatosensory map expansion and altered processing of tactile inputs in a mouse model of fragile X syndrome. Neurobiol Dis. 2016;96:201–15. 27616423 10.1016/j.nbd.2016.09.007

[16] Miceli S, Nadif Kasri N, Joosten J Reduced inhibition within layer IV of Sert knockout rat barrel cortex is associated with faster sensory integration. Cereb Cortex. 2017;27:933–49. 28158484 10.1093/cercor/bhx016 PMC5390402

[17] Pang RD, Wang Z, Klosinski LP Mapping functional brain activation using [14C]-iodoantipyrine in male serotonin transporter knockout mice. PLoS One. 2011;6:e23869. 21886833 10.1371/journal.pone.0023869 PMC3160305

[18] Huang C, Resnik A, Celikel T, Adaptive spike threshold enables robust and temporally precise neuronal encoding. PLoS Comput Biol. 2016;12:e1004984. 27304526 10.1371/journal.pcbi.1004984 PMC4909286

[19] Zhang Z, Jiao YY, Sun QQ Developmental maturation of excitation and inhibition balance in principal neurons across four layers of somatosensory cortex. Neuroscience. 2011;174:10–25. 21115101 10.1016/j.neuroscience.2010.11.045 PMC3020261

[20] Ashby MC, Isaac JTR Maturation of a recurrent excitatory neocortical circuit by experience-dependent unsilencing of newly formed dendritic spines. Neuron. 2011;70:510–21. 21555076 10.1016/j.neuron.2011.02.057 PMC3092126

[21] Cheetham CEJ, Fox K Presynaptic development at L4 to l2/3 excitatory synapses follows different time courses in visual and somatosensory cortex. J Neurosci. 2010;30:12566–71. 20861362 10.1523/JNEUROSCI.2544-10.2010 PMC2962420

[22] Stern EA, Maravall M, Svoboda K Rapid development and plasticity of layer 2/3 maps in rat barrel cortex in vivo. Neuron. 2001;31:305–15. 11502260 10.1016/s0896-6273(01)00360-9

[23] Lo SQ, Sng JCG, Augustine GJ Defining a critical period for inhibitory circuits within the somatosensory cortex. Sci Rep. 2017;7:7271. 28779074 10.1038/s41598-017-07400-8 PMC5544762

[24] Diamond ME, Petersen RS, Harris JA Investigations into the organization of information in sensory cortex. J Physiol Paris. 2003;97:529–36. 15242662 10.1016/j.jphysparis.2004.01.010

[25] Kole K, Komuro Y, Provaznik J Transcriptional mapping of the primary somatosensory cortex upon sensory deprivation. GigaScience. 2017;6:1–6. 10.1093/gigascience/gix081 PMC5965344 29020745

[26] Kole K, Komuro Y, Provaznik J, Supporting data for “Transcriptional mapping of the primary somatosensory cortex upon sensory deprivation.”. GigaScience Database. 2017, 10.1093/gigascience/gix081 PMC5965344 29020745

[27] Kole K, Lindeboom RGH, Baltissen MPA Proteomic landscape of the primary somatosensory cortex upon sensory deprivation. GigaScience. 2017;6:1–10. 10.1093/gigascience/gix082 PMC5632293 29020746

[28] Kole K, Lindeboom RGH, Baltissen MPA Supporting data for “Proteomic landscape of the primary somatosensory cortex upon sensory deprivation.” GigaScience Database. 2017, . 10.1093/gigascience/gix082 PMC5632293 29020746

[29] Martens MB, Celikel T, Tiesinga PHE A developmental switch for hebbian plasticity. PLoS Comput Biol. 2015;11:e1004386. 26172394 10.1371/journal.pcbi.1004386 PMC4501799

[30] Stewart RS, Huang C, Arnett MT, Spontaneous oscillations in intrinsic signals reveal the structure of cerebral vasculature. J Neurophysiol. 2013;109:3094–104. 23554431 10.1152/jn.01200.2011

[31] Kole K, Celikel T Neocortical microdissection at columnar and laminar resolution for molecular interrogation. Curr Protoc Neurosci. 2018;e55 doi:10.1002/cpns.55. 30285322 10.1002/cpns.55

[32] Blanton MG, Lo Turco JJ, Kriegstein AR Whole cell recording from neurons in slices of reptilian and mammalian cerebral cortex. J Neurosci Methods. 1989;30:203–10. 2607782 10.1016/0165-0270(89)90131-3

[33] Margrie TW, Brecht M, Sakmann B In vivo, low-resistance, whole-cell recordings from neurons in the anaesthetized and awake mammalian brain. Pflugers Arch. 2002;444:491–8. 12136268 10.1007/s00424-002-0831-z

[34] Zeldenrust F, de Knecht S, Wadman WJ, Estimating the information extracted by a single spiking neuron from a continuous input time series. Front Comput Neurosci. 2017;11:49. 28663729 10.3389/fncom.2017.00049 PMC5471316

[35] Rapid mutual information calculation using frozen noise injection. https://github.com/DepartmentofNeurophysiology/Analysis-tools-for-electrophysiological-somatosensory-cortex-databank/tree/master/Frozen%20Noise.

[36] Lantyer Ad, Calcini N, Bijlsma A, Supporting data for “A databank for intracellular electrophysiological mapping of the adult somatosensory cortex.”. GigaScience Database. 2018 10.5524/100535. PMC6302958 30521020

[37] Neurodata without borders. https://www.nwb.org. Accessed 5 Oct 2018.

[38] Allen Institute Cell Types database. http://celltypes.brain-map.org. Accessed 5 Oct 2018.

[39] ModelDB database. https://senselab.med.yale.edu/ModelDB/. Accessed 5 Oct 2018.

[40] Ince RAA, Senatore R, Arabzadeh E, Information-theoretic methods for studying population codes. Neural Netw. 2010;23:713–27. 20542408 10.1016/j.neunet.2010.05.008

[41] Quian Quiroga R, Panzeri S Extracting information from neuronal populations: information theory and decoding approaches. Nat Rev Neurosci. 2009;10:173–85. 19229240 10.1038/nrn2578

[42] Collaborative Research in Computational Neuroscience data sharing initiative. https://crcns.org/. Accessed 5 Oct 2018.

