# Supplementary material for: A databank for intracellular electrophysiological mapping of the adult somatosensory cortex
Source: Gigascience. 2018 Dec 6;7(12):giy147. doi: 10.1093/gigascience/giy147 (PMC6302958; doi:10.1093/gigascience/giy147)
Supplement: GIGA-D-18-00318_Original_Submission.pdf [file giy147_giga-d-18-00318_original_submission.pdf]

GigaScience

A databank for intracellular electrophysiological mapping of the adult somatosensory cortex

--Manuscript Draft--

**Manuscript Number:**	GIGA-D-18-00318
**Full Title:**	A databank for intracellular electrophysiological mapping of the adult somatosensory cortex
**Article Type:**	Data Note
**Funding Information:**	Conselho Nacional de Desenvolvimento Científico e Tecnológico (NA) — Ms Angelica da Silva Lantyer<br>Nederlandse Organisatie voor Wetenschappelijk Onderzoek (863.150.25) — Dr Fleur Zeldenrust<br>H2020 European Research Council (660328) — Dr. Tansu Celikel<br>Interreg (122035) — Dr. Tansu Celikel<br>Nederlandse Organisatie voor Wetenschappelijk Onderzoek (824.14.022) — Dr. Tansu Celikel
**Abstract:**	Background: Neurons in the supragranular layers of the somatosensory cortex integrate sensory (bottom-up) and cognitive/perceptual (top-down) information as they orchestrate communication across cortical columns. It has been inferred, based on intracellular recordings from juvenile animals, that supragranular neurons are electrically mature by the fourth postnatal week. However, the dynamics of the neuronal integration in the adulthood is largely unknown. Electrophysiological characterization of the active properties of these neurons throughout adulthood will help to address the biophysical and computational principles of the neuronal integration.<br>Findings: Here we provide a database of whole-cell intracellular recordings from 294 neurons located in the supragranular layers (L2/3) of the primary somatosensory cortex in adult mice (9-45 weeks old) from both sexes (females, N=184; males, N=110). Data include 336 somatic current-clamp (CC) and 515 voltage-clamp (VC) experiments, recorded using a step-and-hold protocol (CC, N=236; VC, N=54), frozen noise injections (CC, N=100) and triangular voltage sweeps (VC, 10 (N=142), 50 (N=157) and 100 ms (N=162)), from regularly spiking (N=161) and fast-spiking neurons (N=78).<br>Conclusions: The data could be used to systematically study the properties of somatic integration, and the principles of action potential generation across sexes and across electrically characterized neuronal classes in adulthood. Understanding the principles of the somatic transformation of postsynaptic potentials into action potentials will shed light onto the computational principles of intracellular information transfer in single neurons and information processing in neuronal networks, helping to recreate neuronal functions in artificial systems.
**Corresponding Author:**	Tansu Celikel, PhD<br>Donders Institute for Brain, Cognition and Behaviour<br>NETHERLANDS
**Corresponding Author Secondary Information:**	
**Corresponding Author's Institution:**	Donders Institute for Brain, Cognition and Behaviour
**Corresponding Author's Secondary Institution:**	
**First Author:**	Angelica da Silva Lantyer
**First Author Secondary Information:**	

Powered by Editorial Manager® and ProduXion Manager® from Aries Systems Corporation

**Order of Authors:**	Angelica da Silva Lantyer
	Niccolò Calcini
	Ate Bijlsma
	Fleur Zeldenrust
	Wim J.J. Scheenen
	Tansu Celikel, PhD
**Order of Authors Secondary Information:**	
**Additional Information:**	
**Question**	**Response**
Are you submitting this manuscript to a special series or article collection?	No
**Experimental design and statistics**<br><br>Full details of the experimental design and statistical methods used should be given in the Methods section, as detailed in our Minimum Standards Reporting Checklist. Information essential to interpreting the data presented should be made available in the figure legends.<br><br>Have you included all the information requested in your manuscript?	Yes
**Resources**<br><br>A description of all resources used, including antibodies, cell lines, animals and software tools, with enough information to allow them to be uniquely identified, should be included in the Methods section. Authors are strongly encouraged to cite Research Resource Identifiers (RRIDs) for antibodies, model organisms and tools, where possible.<br><br>Have you included the information requested as detailed in our Minimum Standards Reporting Checklist?	Yes
**Availability of data and materials**<br><br>All datasets and code on which the	Yes

conclusions of the paper rely must be either included in your submission or deposited in publicly available repositories (where available and ethically appropriate), referencing such data using a unique identifier in the references and in the “Availability of Data and Materials” section of your manuscript.<br><br>Have you have met the above requirement as detailed in our Minimum Standards Reporting Checklist?	

Powered by Editorial Manager® and ProduXion Manager® from Aries Systems Corporation

Manuscript Click here to download Manuscript Lantyer_Datanote.pdf

A Datanote submission to GigaScience

A databank for intracellular electrophysiological mapping of the adult somatosensory cortex

Angelica da Silva Lantyer*, Niccolò Calcini*, Ate Bijlsma*, Fleur Zeldenrust, Wim J. J. Scheenen, Tansu Celikel[CA]

Department of Neurophysiology, Donders Institute for Brain, Cognition and Behaviour, Radboud University, Nijmegen - the Netherlands
* denotes equal contribution; [CA] Corresponding author

E-mail addresses:
{a.lantyer n.calcini a.bijlsma f.zeldenrust w.scheenen t.celikel}@neurophysiology.nl

Keywords (3-10)

Whole-cell intracellular recordings, somatic patch-clamp, current-clamp, voltage-clamp, acute brain slices, adult brain, barrel cortex, frozen noise, big data

Competing interests

The authors declare that they have no competing interests.

Funding

This work was supported by a doctoral fellowship from the National Council for Scientific and Technological Development of Brazil (CNPQ) to ASL, and the grants from the European Commission (Horizon2020, nr. 660328), European Regional Development Fund (MIND, nr. 122035) and the Netherlands Organisation for Scientific Research (NWO-ALW Open Competition, nr. 824.14.022) to TC, and by the Netherlands Organisation for Scientific Research (NWO Veni Research Grant, nr. 863.150.25) to FZ.

Acknowledgements

We would like to thank the members of the Department of Neurophysiology for stimulating discussions and the critical insight on the manuscript.

Abstract (250/250 words)

Background: Neurons in the supragranular layers of the somatosensory cortex integrate sensory (bottom-up) and cognitive/perceptual (top-down) information as they orchestrate communication across cortical columns. It has been inferred, based on intracellular recordings from juvenile animals, that supragranular neurons are electrically mature by the fourth postnatal week. However, the dynamics of the neuronal integration in the adulthood is largely unknown. Electrophysiological characterization of the active properties of these neurons throughout adulthood will help to address the biophysical and computational principles of the neuronal integration.

Findings: Here we provide a database of whole-cell intracellular recordings from 294 neurons located in the supragranular layers (L2/3) of the primary somatosensory cortex in adult mice (9-45 weeks old) from both sexes (females, N=184; males, N=110). Data include 336 somatic current-clamp (CC) and 515 voltage-clamp (VC) experiments, recorded using a step-and-hold protocol (CC, N=236; VC, N=54), frozen noise injections (CC, N=100) and triangular voltage sweeps (VC, 10 (N=142), 50 (N=157) and 100 ms (N=162)), from regularly spiking (N=161) and fast-spiking neurons (N=78).

Conclusions: The data could be used to systematically study the properties of somatic integration, and the principles of action potential generation across sexes and across electrically characterized neuronal classes in adulthood. Understanding the principles of the somatic transformation of postsynaptic potentials into action potentials will shed light onto the computational principles of intracellular information transfer in single neurons and information processing in neuronal networks, helping to recreate neuronal functions in artificial systems.

Data description

The primary somatosensory cortex (S1) encodes time varying haptic information from the mechanoreceptors in the skin, thereby representing a topographical map [1, 2]. Rodents, for example, locate tactile targets in their immediate environment by integrating information across (topographical) whisker representations in the barrel cortex [3], where neurons in each cortical column preferably respond to a single whisker on the contralateral snout [4]. The supragranular layer (cortical layers 2/3, L2/3) of the barrel cortex is the first cortical network that integrates the sensory information across neighboring cortical columns, whiskers and whisk cycles [5–8]. This representation of the whisker contacts undergoes experience dependent changes [9–12] and is altered in animal models of neurodevelopmental disorders [13–15]. Adaptive changes in the synaptic and modulatory drive could powerfully regulate the transformation of postsynaptic responses into action potentials, ultimately controlling how sensory information is transferred between cortical columns and cortical regions [16].

Understanding the principles of neuronal information transfer in the supragranular layer will require systematic analysis of the integrative properties of these neurons. Here we provide a database of 851 experiments collected from 294 supragranular neurons. The database consists of whole-cell intracellular recordings in voltage-clamp (VC) and current-clamp (CC) configurations: while current-clamp somatic measurements bring insight into the properties related to action potential initiation, timing, rate and pattern, voltage-clamp recordings provide information on the voltage-gated ion-channel dynamics. The database is best utilized to address the principles of information transfer in individual neurons (see e.g. [16, 17]) and for electrical classification of adult cortical sensory neurons. It will serve synaptic, systems,

computational and theoretical neuroscientist in search of the principles of information processing, transfer and recovery in neuronal networks. The database is expected to create synergy with 1) the recently completed transcriptome [18, 19] and proteome [20, 21] of the supragranular layers of the barrel cortex, 2) the computational models of the molecular changes that contribute to the maturation of synaptic communication in the same cortical region (e.g [22]), and 3) the high resolution mapping of sensory representations using intrinsic signals in single trial resolution (e.g. [23]), resulting in a multi-scale analysis of the cortical organization, from molecules of chemical communication to network representations.

Methods

Experiments that involve animals were conducted in accordance with the European Directive 2010/63/EU, national regulations in the Netherlands, and international guidelines on animal care and use of animals. Pvalbtm1(cre)Arbr (RRID:MGI:5315557) or Ssttm2.1(cre)Zjh/J mice (RRID:IMSR_JAX:013044) from either sexes (N=65 females, N=40 males, aged 9-45 weeks) were used from the local breeding colonies.

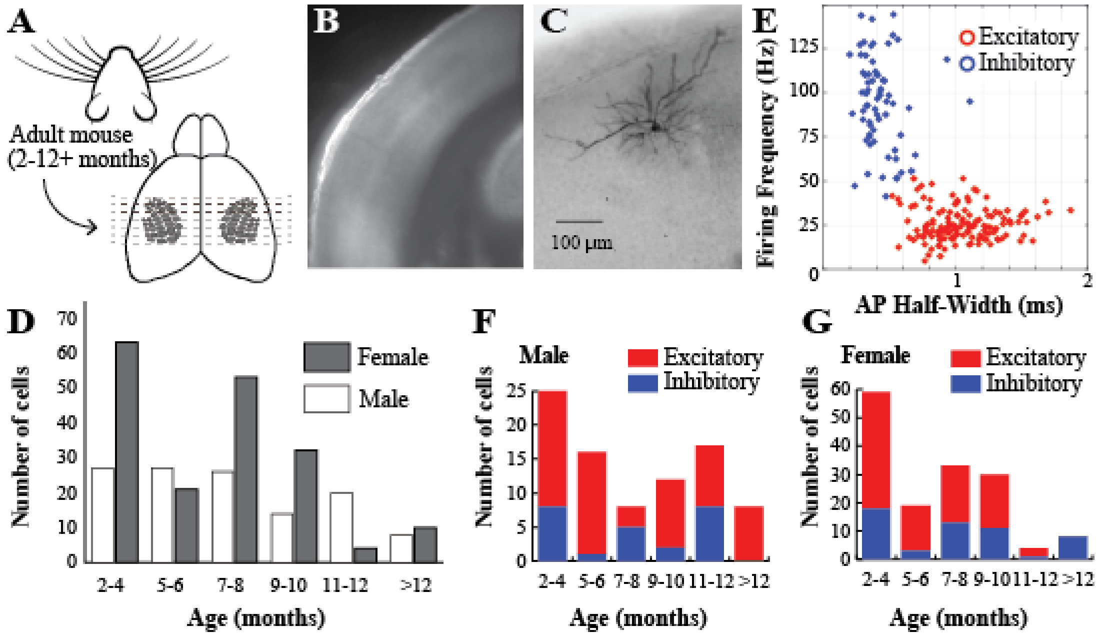

Figure 1. Acute slice preparation. **(A)** Coronal slices (300 micrometer in thickness) were prepared for ex vivo recording from the L2/3 neurons in the mouse primary somatosensory cortex, barrel cortex subregion. **(B)** A low magnification view of the slice in 4x. **(C)** A representative neuron, intracellularly filled with biocytin, and visualized with DAB staining. **(D)** Distribution of the 294 neurons in this database across males (37.4%) and females (62.6%) as well as age of the animal. **(E)** Classification of the neurons presumed inhibitory and excitatory populations based on firing frequency and action potential half-width (see Methods for details). **(F)** The distribution of cells across cell type and ages.

The mice were anaesthetised with Isoflurane (1.5 ml/mouse) before the tissue was extracted. The depth of anaesthesia was assessed by pinch withdrawal before ice-cold dissection solution containing (in mM) 108 choline chloride, 3 KCl, 26 $NaHCO_3$, 1.25 $NaH_2PO_4.H_2O$, 25 $Glucose.H_2O$, 1 $CaCl_2.2H_2O$, 6 $MgSO_4.7H_2O$, 3 sodium pyruvate was perfused intracardially. The brain was removed after decapitation and sliced coronally (300 micrometer in thickness) in the same ice-cold perfusion medium. The slices were then transferred to a chamber containing aCSF (in mM): 120

NaCl, 3.5 KCl, 10 Glucose.H_2O, 2.5 $CaCl_2$.2H_2O, 1.3 $MgSO_4$.7H_2O, 25 $NaHCO_3$, 1.25 NaH_2PO_4.H_2O, aerated with 95% O_2/ 5% CO_2 at 37□C. After 30 minutes, the slices were transferred to room temperature before whole-cell electrophysiological recordings started.

Whole-cell recordings

Slices were continuously oxygenated and perfused with aCSF during recordings. The barrel cortex was localized and cells of interest in the supragranular layers were patched under 40x magnification. Patch clamp electrodes were pulled from glass capillaries (1.00 mm (external diameter), 0.50 mm (internal diameter), 75 mm (length), GC100FS-7.5, Harvard Apparatus) with a P-2000 puller (Sutter Instrument, USA) and used if their initial resistance were between 5 and 10 MOhm. They were filled with intracellular solution containing (in mM) 130 K-Gluconate, 5 KCl, 1.5 $MgCl_2$.6H_2O, 0.4 Na3GTP, 4 Na2ATP, 10 HEPES, 10 Na-phosphocreatine, 0.6 EGTA, and the pH was set at 7.22 with KOH. Current-clamp and voltage-clamps recordings were performed as described before [24], [25] and included four stimulus protocols (Figure 2).

Current-clamp protocol

After establishing the current-clamp configuration, the resting membrane potential was set to -70 mV by direct somatic current injections, as required. The step-and-hold stimulation protocol included 10 steps of 500 ms long depolarization pulses (step size: 5, 10, 20, 40 or 60 pA) with an inter-sweep-interval of 6.5 s. The stimulus train was repeated 3 times with a 20 s interval. The frozen-noise (FN) stimulation protocol involved somatic injection of current that approximates the output

of an artificial neural network of 1000 neurons, firing Poisson spike trains in response to a 'hidden state' (see [26] for details, and https://github.com/DepartmentofNeurophysiology/Analysis-tools-for-electrophysiological-somatosensory-cortex-databank/tree/master/Frozen%20Noise on how to generate the frozen noise input and analyze the data).

Voltage-clamp protocol

The voltage-clamp stimulation protocols included step-and-hold and sawtooth (triangular) pulse injections (Figure 2). In both protocols the membrane potential was clamped at -70 mV prior to somatic depolarization. In the step-and-hold protocols, 14 incremental steps of depolarizing pulses (10 mV/each) were delivered for a period of 250 ms with an interval of 20 s. Sawtooth pulses (range: -70 to 70 mV) were delivered at three frequencies (5, 10, 50 Hz) and consisted of five triangular pulses with peak-to-peak (P2P) distances of 200, 100, 20 ms, respectively. Each trial was repeated twice with 20 s interval.

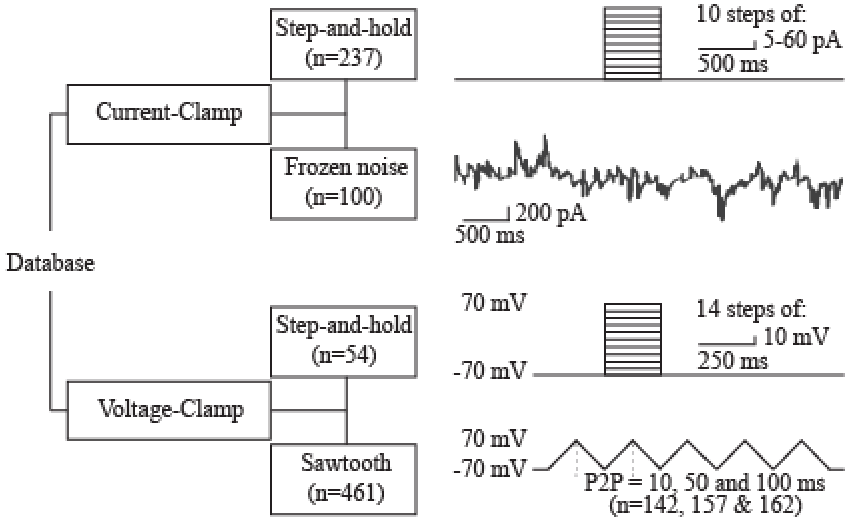

Figure 2. Experimental protocols and the hierarchical organization of the database. The data is available online at https://goo.gl/8hbeZ7

Data organization

Files in ".mat" (MATLAB) format containing the original traces from each experiment are organized in folders separated by the structure described in Figure 2. Metadata including the date and number experiments, experimenter's initials, sex, age, experiment protocol, cell type and animal number are included in a tabulated format (.xlsx, Microsoft Excel). The experiments are named as date_prefix_experiment number_protocol number.

The current clamp data (see "Current Clamp" folder) contains two subfolders, "Step Protocol" and "Frozen Noise". Step Protocol data includes two channels (voltage and current), each of which includes two columns (timestamp and voltage/current values in volt and amp, respectively) for each repetition. Users can visualize both the current injected to clamp the soma and the observed voltage response. Data from each stimulus condition is saved under a separate variable starts with "Trace_a_b_c_d" and includes information about a) cell and experiment ID, b) data type, c) sweeps in each dataset, and d) channels.

The "Frozen Noise" subfolder contains the voltage trace (i.e. neuronal response to the injected frozen noise), hidden state (activity in the modeled network, see [26] for details) and the injected current trace. In addition a Matlab "struct" variable named "settings" is provided. Settings provide metadata under following "fields": condition, experimenter, baseline (membrane potential value (in mV) at which the cell is kept with the baseline current injection), amplidute_scalind (the scaling factor used to translate the output of the neural network in pA value), tau (the time constant that defines the average switching speed of the hidden state), mean_firing_rate (of the artificial neurons), sampling_rate (the acquisition rate (in kHz)), duration (in ms),

FLAG_convert_to_amphere (a binary value that is 1 if the output was converted into Ampere), and cell_type (excitatory vs inhibitory).

The voltage clamp folder includes two subfolders: VC Step (voltage step-and-hold) and VC Sawtooth, the latter containing 3 subfolders with recordings from experiments with triangular sweeps at 3 frequencies (5, 10 or 50 Hz). Data in the Voltage Clamp folder is organized similarly to the Current Clamp folder, and variable naming follows the formatting rules described above.

Cell type classification

K-means clustering (cluster count=2; number of repetition=10) was performed to classify neurons into a cell class, i.e. presumed inhibitory or excitatory, using current clamp step-and-hold recordings. The clustering was based on the maximum firing rate reached during the current step injections and on the mean spike half-width.

Re-use potential

The dataset is rich in information regarding current versus voltage dynamics in adult cortical neurons. The independent variables in the database are the sex and age of the animal. While current-clamp experiments provide information about sub- and suprathreshold voltage dynamics, the voltage-clamp experiments are informative about the ionic conductances that lead to activation or inactivation of neurons.

In the step-and-hold current clamp experiments, the voltage responses can be quantified using subthreshold (e.g. amplitude, latency, duration of the postsynaptic potential) and suprathreshold (e.g. interspike interval adaptation, spike count, spike half-width) responses to somatic current injection (Figure 3). Because multiple stimuli

with incrementally increasing current intensities are delivered, cellular responses can be mapped onto stimulation intensities, allowing users to study input/output curves for the parameters of interest.

Action potentials can be studied both in terms of their shape (e.g. waveform, rise and decay slope, amplitude of the positive and negative peaks, half-width of spike) and temporal response properties (that allow quantification of the rate and timing of action potentials during synaptic activation). Since adaptation to the sustained current injection is commonly used as a criterion to classify neurons, the data provides an inclusive database for the electrical classification of adult neurons, creating synergy with other publicly available databases, e.g. Neurodata Without Borders (https://www.nwb.org) and the Cell Type database (http://celltypes.brain-map.org) of the Allen Institute. The data can be used independently or in the context of computational models of neural networks, a broad selection of which can be found in the ModelDB database (https://senselab.med.yale.edu/ModelDB/).

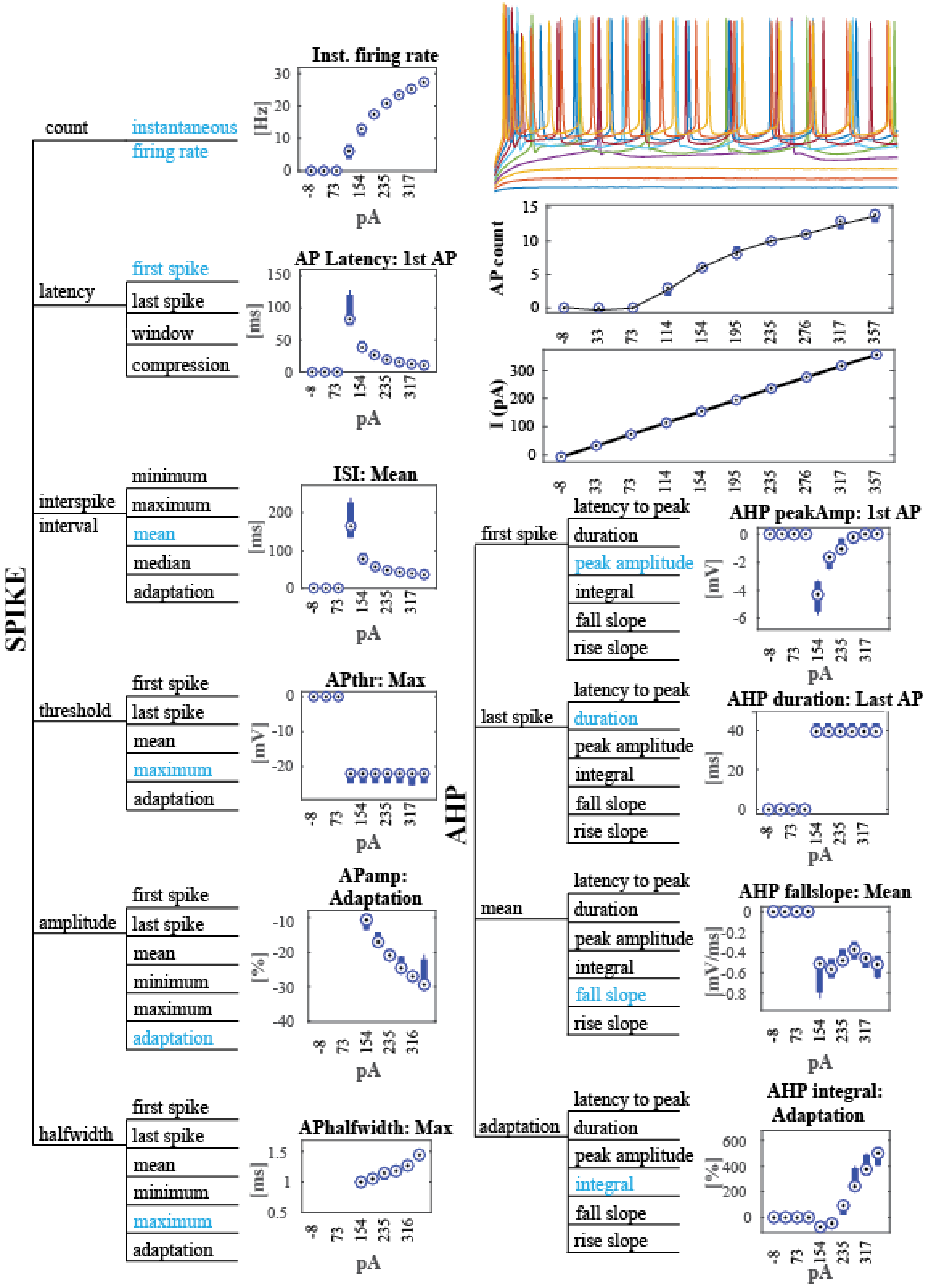

Figure 3. Electrical characterization of the spiking response in current-clamp experiments. The parameter space is shown as a hierarchical tree. Variables shown in blue are used for the data displays. AHP = afterhyperpolarization. All characteristics are measured relative to the stimulus amplitude (the current injected, in pA). Membrane potential traces on the top-right are the responses to incremental current injections, superimposed on top of each other. The data below the raw traces represents the number of action potentials and the amplitude of the injected current across the 10 step-and-hold stimuli in this experiment (Filename: 170130_AL_133).

In addition to sustained somatic depolarization, the current-clamp database also includes “frozen noise” injections, during which a time-varying current was injected into the recorded neuron. The injected current was generated using an artificial neural network (see [26] for details) of ~1000 neurons, each one firing spike trains from an inhomogeneous Poisson process, responding to a binary hidden state which represents the presence or absence of an external stimulus. The activity of all the neurons in the artificial network is integrated and the resulting current is corrected for the baseline current required to keep the patched neuron at -70mV. This summed current is injected to the patched soma. A major utility the frozen noise protocol is to quantify the information transfer properties of single neurons. Compared to other metrics of neuronal information transfer [16, 27, 28], this approach enables bias-free quantification of information with a short (~6 min) stimulation protocol [26].

In the database, experimental data obtained from our frozen noise protocol include the recorded membrane potential voltage, the hidden state and the current injected to the neurons. Thus, the user can perform forward and reverse modeling to predict the neuronal response, and to study neuronal dynamics in the adult neocortex.

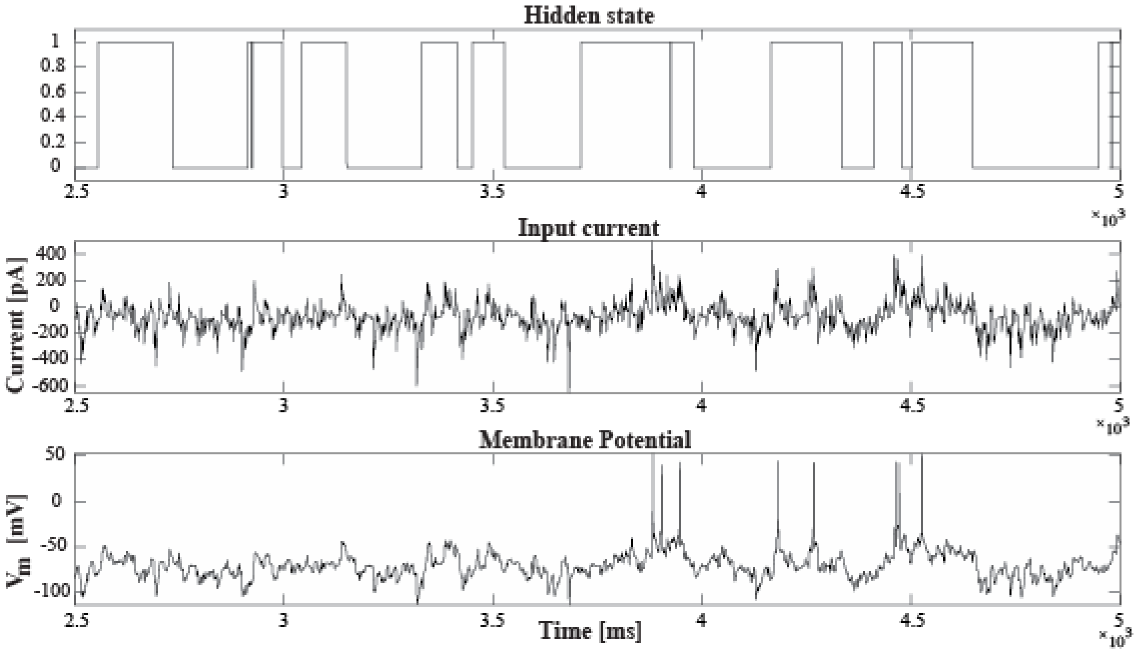

Figure 4. Frozen noise injection in current-clamp configuration. Representative recording from a single neuron (experiment 171207_NC_146). Top row: Binary representation of the hidden state that forms the input to an artificial neural network with 1000 point neurons, firing action potentials following an inhomogeneous Poisson process (see [26] for details). Middle row: the synaptic current generated by the artificial network that was injected into the recorded neuron. Bottom row: the membrane potential response of the recorded neuron.

Going beyond the voltage dynamics in the adult neurons, the database also provides insight into the ionic currents that flow through the membrane. With the triangle shaped VC-Saw protocol (Figure 5) it is possible to measure the activation threshold of the currents flowing through the membrane by looking at when a deviation occurs from the expected sawtooth shape. Additionally, it is possible to compute: amplitudes and latencies of the events, peaks half-widths, percentage difference between consecutive events and the total number of events in each sweep. Other features could also be extracted from the dataset depending on the researchers interests.

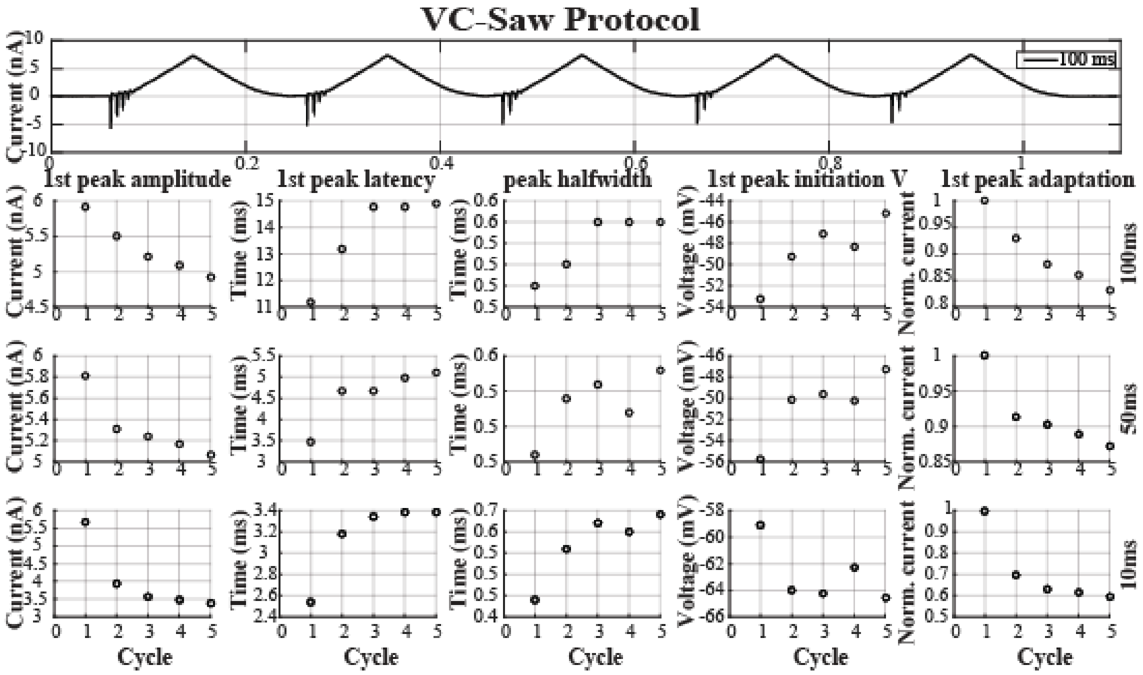

Figure 5. Voltage-clamp sawtooth protocol. Top row: Current trace from a representative experiment (180412_AB_53_ST). Figurines, left to right, are measurements of first peak amplitude, first peak latency, half width of the first inward current, membrane potential at inward current is initiated, and the adaptation of the first event amplitude across the 5 (triangle) cycles. Data in the bottom three row are from three different sawtooth speeds (10/50/100 ms, corresponding to 100/20/10 Hz stimulation). The five points in each figurine are calculated from the first inward current in each (triangle) cycle.

The current-voltage relationship was measured with voltage-clamp steps (Figure 6), which could be used to produce an I/V curve. The peak amplitude, latency, and peak half width can be extracted for the inward currents observed during the sustained depolarization of the soma.

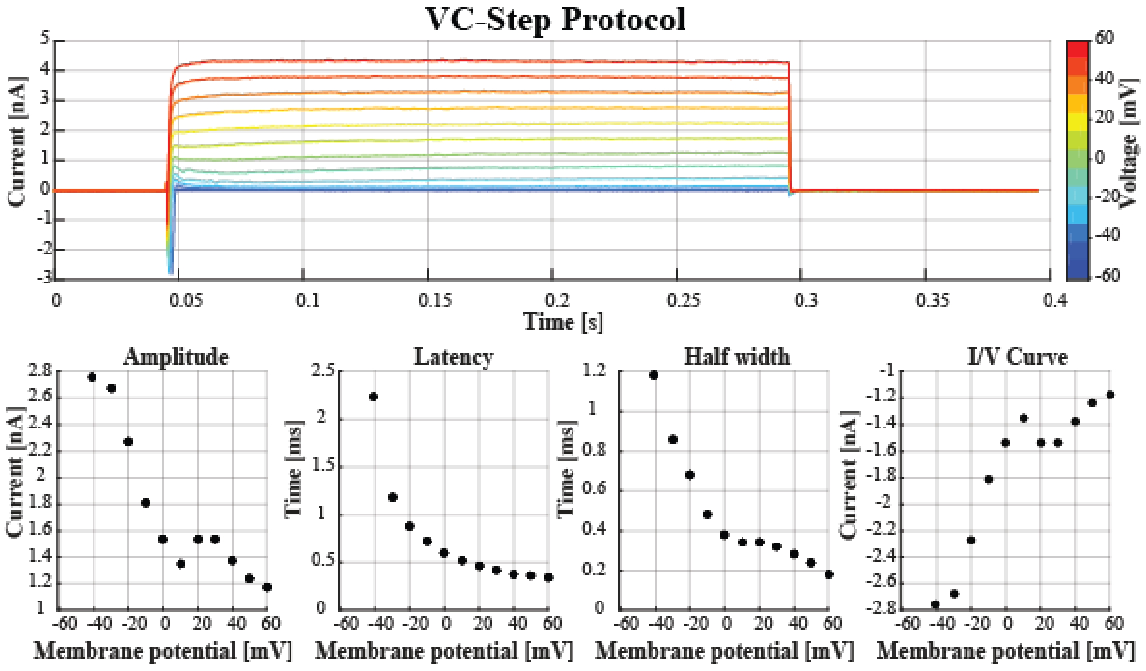

Figure 6. Step-and-hold protocol in voltage-clamp preparation. The main panel shows data from a representative experiment (170915_AB_5_VC). Every other figurines shows one of the analyzed features. Every voltage step is analyzed.

Application scenarios

From the recordings available in this database, it is possible to visualize the membrane properties regarding the current and voltage dynamics and investigate the somatic integration from single neuron active measurements.

Network development is based on processes of self-organization that are highly dependent on plasticity [29]. Such plasticity is not be limited to early life development. From the voltage-clamp and current-clamp experiments, it is possible to infer the biophysical properties characteristics of the layer 2/3 pyramidal neurons of the adult somatosensory cortex. The focus on adult neurons brings a new perspective to the study of the membrane properties, as data from this developmental age are still scarce. The dynamics of electrical properties of the membrane can be accessed as a function of different developmental time points and/or sex. Another possibility is to cluster the

neurons according to their functional characterization and use the recorded data as virtual neurons for dynamic-clamp experiments.

In a computational approach, spike properties from the data here described could be used for modeling small and large scale network activity, facilitating the study of neuronal circuits and providing a basis for high-dimensional or multimodal clustering. In particular, the heterogeneity of neuronal properties can be assessed using this dataset [30]. Moreover, applying the principles of information transfer and recovery to the data might help recreate neuronal functions in artificial systems.

Limitations

Neurons in this dataset originate from both excitatory and inhibitory neurons, however there is no anatomical characterization of the neuron type studied. The database is focused on Layer 2/3 of the somatosensory cortex as a model region and does not allow the study of neuronal information processing across different cortical regions in isolation. However, the user might consider comparing data across different regions and species by utilizing the other publicly available databases, e.g. Neurodata Without Borders (https://www.nwb.org), the Cell Type database (http://celltypes.brain-map.org) of the Allen Institute and the Collaborative Research in Computational Neuroscience data sharing initiative (https://crcns.org/).

List of abbreviations

aCSF - artificial cerebrospinal fluid

AP - action potential

AHP - afterhyperpolarization peak

CC - current clamp

FN - frozen noise

ISI - inter-spike interval

I/V - current/voltage

L2/3 - cortical layer 2/3

ST - sawtooth

S1 - primary somatosensory cortex

VC - voltage clamp

Authors contributions

ASL, NC, AT performed experiments; AB, FF, TC wrote data analysis routines; FF, WJJS, TC supervised the experiments.

References

1. Kole K, Scheenen W, Tiesinga P, Celikel T. Cellular diversity of the somatosensory cortical map plasticity. Neurosci Biobehav Rev. 2018;84:100–15. doi:10.1016/j.neubiorev.2017.11.015.

2. Diamond ME, Petersen RS, Harris JA. Learning through maps: functional significance of topographic organization in primary sensory cortex. J Neurobiol. 1999;41:64–8. doi:10.1002/(SICI)1097-4695(199910)41:1<64::AID-NEU9>3.0.CO;2-N.

3. Celikel T, Sakmann B. Sensory integration across space and in time for decision making in the somatosensory system of rodents. Proc Natl Acad Sci USA. 2007;104:1395–400. doi:10.1073/pnas.0610267104.

4. Van der Loos H, Woolsey TA. Somatosensory cortex: structural alterations following early injury to sense organs. Science. 1973;179:395–8.

5. Voigts J, Herman DH, Celikel T. Tactile object localization by anticipatory whisker motion. J Neurophysiol. 2015;113:620–32. doi:10.1152/jn.00241.2014.

6. Carvell GE, Simons DJ. Task- and subject-related differences in sensorimotor behavior during active touch. Somatosens Mot Res. 1995;12:1–9.

7. Voigts J, Sakmann B, Celikel T. Unsupervised whisker tracking in unrestrained behaving animals. J Neurophysiol. 2008;100:504–15. doi:10.1152/jn.00012.2008.

8. Carvell GE, Simons DJ. Effect of whisker geometry on contact force produced by vibrissae moving at different velocities. J Neurophysiol. 2017;118:jn.00046.2017. doi:10.1152/jn.00046.2017.

9. Allen CB, Celikel T, Feldman DE. Long-term depression induced by sensory deprivation during cortical map plasticity in vivo. Nat Neurosci. 2003;6:291–9. doi:10.1038/nn1012.

10. Celikel T, Szostak VA, Feldman DE. Modulation of spike timing by sensory deprivation during induction of cortical map plasticity. Nat Neurosci. 2004;7:534–41. doi:10.1038/nn1222.

11. Foeller E, Celikel T, Feldman DE. Inhibitory sharpening of receptive fields contributes to whisker map plasticity in rat somatosensory cortex. J Neurophysiol. 2005;94:4387–400. doi:10.1152/jn.00553.2005.

12. Clem RL, Celikel T, Barth AL. Ongoing in vivo experience triggers synaptic metaplasticity in the neocortex. Science. 2008;319:101–4. doi:10.1126/science.1143808.

13. Juczewski K, von Richthofen H, Bagni C, Celikel T, Fisone G, Krieger P. Somatosensory map expansion and altered processing of tactile inputs in a mouse model of fragile X syndrome. Neurobiol Dis. 2016;96:201–15. doi:10.1016/j.nbd.2016.09.007.

14. Miceli S, Nadif Kasri N, Joosten J, Huang C, Kepser L, Proville R, et al. Reduced

Inhibition within Layer IV of Sert Knockout Rat Barrel Cortex is Associated with Faster Sensory Integration. Cereb Cortex. 2017;27:933–49. doi:10.1093/cercor/bhx016.

15. Pang RD, Wang Z, Klosinski LP, Guo Y, Herman DH, Celikel T, et al. Mapping functional brain activation using [14C]-iodoantipyrine in male serotonin transporter knockout mice. PLoS ONE. 2011;6:e23869. doi:10.1371/journal.pone.0023869.

16. Huang C, Resnik A, Celikel T, Englitz B. Adaptive spike threshold enables robust and temporally precise neuronal encoding. PLoS Comput Biol. 2016;12:e1004984. doi:10.1371/journal.pcbi.1004984.

17. Diamond ME, Petersen RS, Harris JA, Panzeri S. Investigations into the organization of information in sensory cortex. J Physiol Paris. 2003;97:529–36. doi:10.1016/j.jphysparis.2004.01.010.

18. Kole K, Komuro Y, Provaznik J, Pistolic J, Benes V, Tiesinga P, et al. Transcriptional mapping of the primary somatosensory cortex upon sensory deprivation. Gigascience. 2017.

19. Kole K, Komuro Y, Provaznik J, Pistolic J, Benes V, Tiesinga P. Supporting data for "Transcriptional mapping of the primary somatosensory cortex upon sensory deprivation." GigaDB. 2017.

20. Kole K, Lindeboom RGH, Baltissen MPA, Jansen PWTC, Vermeulen M, Tiesinga P, et al. Proteomic landscape of the primary somatosensory cortex upon sensory deprivation. Gigascience. 2017;6:1–10. doi:10.1093/gigascience/gix082.

21. Kole K, Lindeboom RGH, Baltissen MPA, Jansen PWTC, Vermeulen M, Tiesinga P, et al. Supporting data for "Proteomic landscape of the primary somatosensory cortex upon sensory deprivation." GigaDB. 2017.

22. Martens MB, Celikel T, Tiesinga PHE. A developmental switch for hebbian plasticity. PLoS Comput Biol. 2015;11:e1004386. doi:10.1371/journal.pcbi.1004386.

23. Stewart RS, Huang C, Arnett MT, Celikel T. Spontaneous oscillations in intrinsic signals reveal the structure of cerebral vasculature. J Neurophysiol. 2013;109:3094–104. doi:10.1152/jn.01200.2011.

24. Blanton MG, Lo Turco JJ, Kriegstein AR. Whole cell recording from neurons in slices of reptilian and mammalian cerebral cortex. J Neurosci Methods. 1989;30:203–10.

25. Margrie TW, Brecht M, Sakmann B. In vivo, low-resistance, whole-cell recordings from neurons in the anaesthetized and awake mammalian brain. Pflugers Arch. 2002;444:491–8. doi:10.1007/s00424-002-0831-z.

26. Zeldenrust F, de Knecht S, Wadman WJ, Denève S, Gutkin B. Estimating the Information Extracted by a Single Spiking Neuron from a Continuous Input Time Series. Front Comput Neurosci. 2017;11:49. doi:10.3389/fncom.2017.00049.

27. Ince RAA, Senatore R, Arabzadeh E, Montani F, Diamond ME, Panzeri S. Information-theoretic methods for studying population codes. Neural Netw.

2010;23:713–27. doi:10.1016/j.neunet.2010.05.008.

28. Quian Quiroga R, Panzeri S. Extracting information from neuronal populations: information theory and decoding approaches. Nat Rev Neurosci. 2009;10:173–85. doi:10.1038/nrn2578.

29. Sporns O. Contributions and challenges for network models in cognitive neuroscience. Nat Neurosci. 2014;17:652–60. doi:10.1038/nn.3690.

30. Marder E, Taylor AL. Multiple models to capture the variability in biological neurons and networks. Nat Neurosci. 2011;14:133–8. doi:10.1038/nn.2735.

Metadata

Click here to access/download
Supplementary Material
Lantyer_Metadata.xlsx

Cover Letter

Click here to download Personal Cover Lantyer_Cover letter.pdf

Donders Institute
for Brain, Cognition and Behaviour

Department of Neurophysiology

Professor Tansu Celikel, Ph.D.
Founding Chair, Department of Neurophysiology

Founding Director,
Dutch/German Graduate School in Bionics

Speaker, Donders Institute
Theme: Brain Networks and Neuronal Communication

Heyendaalseweg 135
6525 AJ, Nijmegen
the Netherlands

(+31) (0) 24 365 2702
www.ru.nl/neurophysiology
www.neurophysiology.nl
t.celikel@donders.ru.nl

August 23rd, 2018

Dear Editor

Please accept the accompanying manuscript, entitled "A databank for intracellular electrophysiological mapping of the adult somatosensory cortex" as a Datanote submission to Gigascience.

Developments in high-throughput data collection methods have revolutionized many branches of Neuroscience, however an extensive database for intracellular electrophysiological recordings is not yet publicly available. In this Datanote we open source 851 datasets collected from 294 adult cortical neurons across both sexes. We believe, the database will provide a long sought-after resource for cellular, systems, computational, biophysical, and theoretical neuroscientists to study the functional properties of electrical signaling. This dataset will also create direct synergy with the recently published transcriptome and proteome of the somatosensory cortex, the same region we have collected the electrophysiological data, whose datasets we made publicly available via Gigascience [1, 2].

Given the novelty of this dataset (intracellular electrophysiological studies are typically published with ~20 observations), its suitability for high-dimensional analysis of the neuronal dynamics, and re-use potential across several branches of neuroscience, we are encouraged to submit this database to your consideration for Gigascience.

Sincerely yours,

T. Celikel

Tansu Celikel, on behalf of the authors

References

[1] Kole K, Komuro Y, Provaznik J, Pistolic J, Benes V, Tiesinga P, Celikel T. Transcriptional mapping of the primary somatosensory cortex upon sensory deprivation. Gigascience. 2017. Gigascience. 2017; 6:1–6. doi:10.1093/gigascience/gix081.

[2] Kole K, Lindeboom RGH, Baltissen MPA, Jansen PWTC, Vermeulen M, Tiesinga P, Celikel T. Proteomic landscape of the primary somatosensory cortex upon sensory deprivation. Gigascience. 2017; 6:1–10. doi:10.1093/gigascience/gix082.
