# Supplementary material for: A databank for intracellular electrophysiological mapping of the adult somatosensory cortex
Source: Gigascience. 2018 Dec 6;7(12):giy147. doi: 10.1093/gigascience/giy147 (PMC6302958; doi:10.1093/gigascience/giy147)
Supplement: Reviewer_3_Original_Submission_(Attachment).pdf [file giy147_reviewer_3_original_submission_(attachment).pdf]

GIGA-D-18-00318

A databank for intracellular electrophysiological mapping of the adult somatosensory cortex
Angelica da Silva Lantyer; Niccolò Calcini; Ate Bijlsma; Fleur Zeldenrust; Wim J.J. Scheenen; Tansu Celikel, PhD

GigaScience

This is a comprehensive paper that provides data from 851 experiments collected from 294 cells recorded from the L2/3 of the somatosensory cortex.
The data presented and provided by the authors are adequate for the purposes specified by the authors. While it is a little disappointing that the authors do not draw any conclusions from their massive dataset, it is nevertheless a significant resource for experimental and computational neuroscientists.

I do have a few minor points that need to be addressed:
1. *"it has been inferred based on intracellular recordings from juvenile animals that supragranular neurons are electrically mature by the fourth postnatal week. However the dynamics of the neuronal integration in the adulthood is largely unknown."*

This is a very strong statement to make. If the literature is sparse, it might be worthwhile acknowledging the little knowledge that is known.

2. Further, it will be of interest to the reader to understand the approach used by the authors that is different from those used in juvenile animals. Given the comprehensive nature of the work, any methodological insights would be valuable for the experimental neuroscience community and an extended discussion of the same will be helpful.

3. Figure 2 legend: Links to a google drive folder does not work. Will need to be rectified prior to publication. I was able to access this via the ftp site.

4. Looking at the list of experiments, were all data that had the same date acquired from slices dissected from the same mouse or were multiple mice used.

5. Fig. 1C shows representative biocytin staining image. How many cells in the database were stained? IF available, making this data available, for however many cells that were analyzed would be very valuable for correlating electrophysiology data with morphology.

6. Figure. 3 has several abbreviations/ short phrases that need be to defined in the figure legend.
