# Supplementary material for: A databank for intracellular electrophysiological mapping of the adult somatosensory cortex
Source: Gigascience. 2018 Dec 6;7(12):giy147. doi: 10.1093/gigascience/giy147 (PMC6302958; doi:10.1093/gigascience/giy147)
Supplement: Response_to_Reviewer_Comments_Revision_1.pdf [file giy147_response_to_reviewer_comments_revision_1.pdf]

Author's Response To Reviewer Comments

Close

Please refer to the attached pdf document for the response to the reviewers.

Close
