# Supplementary material for: A databank for intracellular electrophysiological mapping of the adult somatosensory cortex
Source: Gigascience. 2018 Dec 6;7(12):giy147. doi: 10.1093/gigascience/giy147 (PMC6302958; doi:10.1093/gigascience/giy147)
Supplement: Response_to_Reviewer_Comments_Revision_2.pdf [file giy147_response_to_reviewer_comments_revision_2.pdf]

Author's Response To Reviewer Comments

Close

We would like to thank the Reviewers for their careful consideration. In this second revision of the manuscript, there was only one remaining request. Reviewer #1 noted that "Concerning the hum noise present in the recordings, the authors could also add a statement that a certain ratio of the recordings contains hum noise…". Accordingly we included the following sentence on Page 6: "The AC mains (hum) noise (max peak-to-peak amplitude 0.2 mV) that exists in a subset (~4%) of the recordings was not filtered."

We hope this version of the manuscript is suitable for publication as DataNote in Gigascience.

Kind regards
The authors

Close
