# Supplementary material for: A databank for intracellular electrophysiological mapping of the adult somatosensory cortex
Source: Gigascience. 2018 Dec 6;7(12):giy147. doi: 10.1093/gigascience/giy147 (PMC6302958; doi:10.1093/gigascience/giy147)
Supplement: Reviewer_1_Report_(Original_Submission) -- Janos Fuzik — 9/7/2018 Reviewed [file giy147_reviewer_1_report_(original_submission).pdf]

Reviewer Report

Title: **A databank for intracellular electrophysiological mapping of the adult somatosensory cortex**

Version: **Original Submission** **Date:** 9/7/2018

Reviewer name: **Janos Fuzik**

Reviewer Comments to Author:

The authors provide a database of 294 whole-cell recorded neurons located in Layer2/3 of the S1 cortex. The authors carried out both current-clamp and voltage-clamp recordings on the probed neurons and sampled the cells across 6 age groups and both sexes.
The effort is constructive and data sharing serves the good of scientific community.

I think the language of the manuscript is clear and easy to read, the explanation of methods is clear.

Major concerns:

- Recording quality:
I used the sample data provided by the authors to get an insight into the quality of the whole-cell recordings. I checked 7 Current-clamp recordings and found major errors in the recordings with only opening up 7 recordings. I give some of the examples:

170220_AL_191_CC.mat Trace_1_4_9_2 : The holding potential of the recording is between -80 and -85 mV, which is far from the -70 mV described in the methods section. Since the manuscript refers to providing a solid data set to analyze current-voltage relations of L2/3 neurons, -70mV should be kept consistent, otherwise the rheobase current is already wrong, and already biasing the proper clustering of the data.

1703_AB_258_CC.mat Trace_2_4_9_2 : The action potential threshold (APthr) is around +10 mV. This is unseen in L2/3 and I believe, everywhere in the neocortex, since the activation of voltage-gated sodium channels is around -30 mV. The baseline of the recording also shows a periodic waving noise, which arises from the huge access resistance of the recording, also accompanied with the lack of pipette capacitance compensation which can cause parasitic oscillations. In other words, the recording capillary has a high resistance and the access is bad, and not bridge-balanced. This explains why the whole trace is shifted up with APthr of the spikes above zero.

170328_AL_238_CC.mat Trace5_1_10_2 : The holding potential is thus closer to the value stated in the methods, it is -76mV, it is still not -70 mV, which still changes the readout of parameters.

161208_AL_106_CC.mat Trace_2_3_4_2 : Here the holding voltage is -45 mV, the contact is bad to the cell, and noisy combined with a huge access resistance which brings up the APthr again similarly to the earlier example to +5 mV and on Trace_2_3_10_2 it is already +15 mV which is a pure recording error.

In my opinion these are major errors in the whole-cell recordings and the data quality does not seem to be controlled with data discarding criteria. I randomly checked a few cells and I immediately came across major errors thus I think the data provided by the author does not serve the scientific community with sharing it to them.

- Calssification: besides the goal of the paper is not to provide a new classification, the authors make a classification. Clustering classification of neurons based on 2 parameters is not satisfactory. It is enough to think to somatostatin (SST) neurons which has very similar spike width/half-width to that of pyramid cells. Spike width as a parameter would rather separate parvalbumin (PV) expressing fast spiker neurons, vasointestinal peptide (VIP) expressing ones and the rest. This is also represented by Figure1 (E) where two larger clusters are connected with some continuum. That is probably a mix of the mentioned cell types but the major neuronal types of L2/3, the pyramid cells, the PV, SST and VIP neurons are clearly segregating

based on their intrinsic properties and spiking patterns, that is not novelty but the presented analysis rather masks this fact than recapitulates it.

Also, the average of spike width is not correct. It is not specified which spikes are averaged, is it spikes from double threshold or triple trace, or else.. But even if it was specified it is strongly distorting the classification. I give you an example recording:

170509_AB_310_CC.mat Trace_3_3_10_2 : This cell type on this trace has the first spike approx ~40% the width of that of the last spike on the trace, thus averaging masks this parameter and the cell can be classified as a pyramid while not being one for sure based on the trace. Also VIP interneurons are starting their firing pattern with a burst which has different frequency than the rest of the trace, meaning that the average of frequency is a similarly masking way of analyzing electrical signature of neurons.

- Statistical power of the data: the authors carried out a large number of recordings and did a huge amount of work.

Considering that 294 cells fall into 6 age ranges (without read out the exact numbers) would roughly give ~50 neurons per age group and that is further divided to 2 sexes gives ~15 cells male and ~35 cells females (~35% and ~65% from males and females) and that is further divided to pyramid cells, and at least 3 major interneuron types (not mentioning that all of them has multiple already identified types) leaves the user of the data with very few cells each.

In case if we consider a smaller resolution just with 2 age groups of "younger" 2-6 months and the "older" >6 months mice, we would have ~150-150 cells. These would be divided to the sexes roughly ~50 cells males and ~100 cells females, and these could be divided to the neuronal types of pyramid, PV, SST, VIP and subtypes of these, than we could have a ~12 cells/type in males and about ~25cells/type in females. These fast estimations are just roughly done but of course here I presume an equal distribution of cell types which is very improbable since no genetic targeting was used, or no analysis and classification based on a priori knowledge of electrophysiological types was done which could have increased the statistical usefulness of the data through biasing the sampling towards more equal numbered groups.

Most importantly referring to the major concerns about recording quality issues, if we would remove all the cells which should be discarded, we would be left with much less recorded neurons, and even the smaller resolution use of the data to compare sexes and ages which is the main advantage of the data claimed in "Re-use potential" section, would get statistically weak.

Minor concerns

- Figure 1 has a panel (G) but there is no legends to panel (G)

- Figure1 (C) shows a biocytin-filled neuron, but in the manuscript there is no indication of position of the cell recorded or morphology data acquired, thus the figure panel is unnecessary; besides there is nothing mentioned about the DAB protocol in the methods section.

- Methods does not mention the temperature of recordings, which influences the spike width (used for the classification)

- The description of the methods is not complete, the type of patch-clamp amplifier used is not mentioned, which is unusual in a purely electrophysiological study.

- In the data files e.g. 170711_NC_52_CC.mat the protocol is different in length, so the protocol format is not consistent throughout the data set: In this file (and probably in some others) it is 10 000 points before the current step, in other files mentioned in the recording quality issues section it is 40 000 points before the current step which makes the extraction of features more difficult.

Methods

Are the methods appropriate to the aims of the study, are they well described, and are necessary controls included? Choose an item.

Conclusions

Are the conclusions adequately supported by the data shown? Choose an item.

Reporting Standards

Does the manuscript adhere to the journal's guidelines on minimum standards of reporting? Choose an item.

Choose an item.

Statistics

Are you able to assess all statistics in the manuscript, including the appropriateness of statistical tests used? Choose an item.

Quality of Written English

Please indicate the quality of language in the manuscript: Choose an item.

Declaration of Competing Interests

Please complete a declaration of competing interests, considering the following questions:

- Have you in the past five years received reimbursements, fees, funding, or salary from an organisation that may in any way gain or lose financially from the publication of this manuscript, either now or in the future?
- Do you hold any stocks or shares in an organisation that may in any way gain or lose financially from the publication of this manuscript, either now or in the future?
- Do you hold or are you currently applying for any patents relating to the content of the manuscript?
- Have you received reimbursements, fees, funding, or salary from an organization that holds or has applied for patents relating to the content of the manuscript?
- Do you have any other financial competing interests?
- Do you have any non-financial competing interests in relation to this paper?

If you can answer no to all of the above, write 'I declare that I have no competing interests' below. If your reply is yes to any, please give details below.

I declare that I have no competing interests

I agree to the open peer review policy of the journal. I understand that my name will be included on my report to the authors and, if the manuscript is accepted for publication, my named report including any attachments I upload will be posted on the website along with the authors' responses. I agree for my report to be made available under an Open Access Creative Commons CC-BY license (http://creativecommons.org/licenses/by/4.0/). I understand that any comments which I do not wish to be included in my named report can be included as confidential comments to the editors, which will not be published.

I agree to the open peer review policy of the journal

To further support our reviewers, we have joined with Publons, where you can gain additional credit to further highlight your hard work (see: https://publons.com/journal/530/gigascience). On publication of this paper, your review will be automatically added to Publons, you can then choose whether or not to claim your Publons credit. I understand this statement.

Yes Choose an item.
