# Supplementary material for: A databank for intracellular electrophysiological mapping of the adult somatosensory cortex
Source: Gigascience. 2018 Dec 6;7(12):giy147. doi: 10.1093/gigascience/giy147 (PMC6302958; doi:10.1093/gigascience/giy147)
Supplement: Reviewer_1_Report_(Revision_1) -- Janos Fuzik — 10/29/2018 Reviewed [file giy147_reviewer_1_report_(revision_1).pdf]

Reviewer Report

Title: A databank for intracellular electrophysiological mapping of the adult somatosensory cortex

Version: Revision 2 Date: 10/29/2018

Reviewer name: Janos Fuzik

Reviewer Comments to Author:

The authors submitted a corrected version of their manuscript: "A databank for intracellular electrophysiological mapping of the adult somatosensory cortex". The effort of the authors to improve the dataset is to be appreciated. The statement added about the possible drifts in the holding membrane potential of the characterized and recorded neurons provides the information for putative future users to handle the dataset with care. Setting up criteria for quality control and removing some of the recordings which fall below the threshold of these requirements reflects the authors intention to improve the data. The authors also added 34 recordings to replace the removed ones and further ensure that the provided 'databank' has a potential to be used by e.g. data scientists.

Concerning the hum noise present in the recordings, the authors could also add a statement that a certain ratio of the recordings contains hum noise, this is to further improve the awareness of future users who might use the dataset to filter membrane oscillations from the spike traces (as many physiologists dealing with e.g. neurogliaform cells do, would benefit from being informed about such details).

The change in the classification of the neurons section is satisfactory, naming the neuron categories "presumed inhibitory" and "presumed excitatory" are more proper considering that otherwise the classification is not precise without further immunohistochemical validation, and indeed the journal does not require the analysis of the cells, thus the authors provide a very simple and basic analysis to ensure the future user of the dataset that there are many narrow-spiker cells (putative interneurons) thus also principal and interneurons of some classes were probed in L2/3.

The structure of the dataset spanning multiple age-ranges and both sexes allows for extensive analysis of current-to-voltage relations in L2/3, capitalizing that statistical potential is much stronger using all the data, without further dividing to groups - since cell type composition of age/sex groups is unknown and the unbiased cell collection-provided cell type ratios might be still different in smaller data bins - would give a precise note on how one could use the dataset.

Taken together, with the suggested minor changes being deployed I think the dataset was improved and with capitalizing the strength and the weaknesses with adding the notes suggested to well inform the future user of the data, the changes made are satisfactory so is the manuscript.

Methods

Are the methods appropriate to the aims of the study, are they well described, and are necessary controls included? Choose an item.

Conclusions

Are the conclusions adequately supported by the data shown? Choose an item.

Reporting Standards

Does the manuscript adhere to the journal's guidelines on [minimum standards of reporting?]() Choose an item.

Choose an item.

Statistics

Are you able to assess all statistics in the manuscript, including the appropriateness of statistical tests used? Choose an item.

Quality of Written English

Please indicate the quality of language in the manuscript: Choose an item.

Declaration of Competing Interests

Please complete a declaration of competing interests, considering the following questions:

- Have you in the past five years received reimbursements, fees, funding, or salary from an organisation that may in any way gain or lose financially from the publication of this manuscript, either now or in the future?
- Do you hold any stocks or shares in an organisation that may in any way gain or lose financially from the publication of this manuscript, either now or in the future?
- Do you hold or are you currently applying for any patents relating to the content of the manuscript?
- Have you received reimbursements, fees, funding, or salary from an organization that holds or has applied for patents relating to the content of the manuscript?
- Do you have any other financial competing interests?
- Do you have any non-financial competing interests in relation to this paper?

If you can answer no to all of the above, write 'I declare that I have no competing interests' below. If your reply is yes to any, please give details below.

I declare that I have no competing interests

I agree to the open peer review policy of the journal. I understand that my name will be included on my report to the authors and, if the manuscript is accepted for publication, my named report including any attachments I upload will be posted on the website along with the authors' responses. I agree for my report to be made available under an Open Access Creative Commons CC-BY license (http://creativecommons.org/licenses/by/4.0/). I understand that any comments which I do not wish to be included in my named report can be included as confidential comments to the editors, which will not be published.

I agree to the open peer review policy of the journal

To further support our reviewers, we have joined with Publons, where you can gain additional credit to further highlight your hard work (see: https://publons.com/journal/530/gigascience). On publication of this paper, your review will be automatically added to Publons, you can then choose whether or not to claim your Publons credit. I understand this statement.

Yes Choose an item.
