# Supplementary material for: A databank for intracellular electrophysiological mapping of the adult somatosensory cortex
Source: Gigascience. 2018 Dec 6;7(12):giy147. doi: 10.1093/gigascience/giy147 (PMC6302958; doi:10.1093/gigascience/giy147)
Supplement: Reviewer_2_Report_(Original_Submission) -- Hongdian Yang — 9/14/2018 Reviewed [file giy147_reviewer_2_report_(original_submission).pdf]

**Reviewer Report**

**Title: A databank for intracellular electrophysiological mapping of the adult somatosensory cortex**

**Version: Original Submission** **Date:** 9/14/2018

**Reviewer name: Hongdian Yang**

**Reviewer Comments to Author:**

Lantyer et al. provided a dataset of whole-cell recordings in S1 slices, with both voltage-clamp and current-clamp recordings and different input configurations. Such dataset has the potential to provide a systematic and comprehensive characterization of the intrinsic and response properties of S1 neurons. The results will facilitate computational and modelling studies.

Major concerns:
-The authors did not include the results of post-hoc reconstruction of the recorded neurons for this dataset (but in Fig. 1C they showed a reconstructed cell). Information such as actual recording depth/layer, morphology, and cell type could be vital to interpret whole-cell recordings.
-I wonder why they used PV-Cre and SST-Cre lines for this study but didn't use optogenetics to identify cell types (or with fluorescent markers for post-hoc identification).

Minor concerns:
-Figure legends for several figures (such as 2, 3, 6) are to concise. Fig. 1G is not explained in the legends.

**Methods**

Are the methods appropriate to the aims of the study, are they well described, and are necessary controls included? Choose an item.

**Conclusions**

Are the conclusions adequately supported by the data shown? Choose an item.

**Reporting Standards**

Does the manuscript adhere to the journal's guidelines on minimum standards of reporting? Choose an item.

Choose an item.

**Statistics**

Are you able to assess all statistics in the manuscript, including the appropriateness of statistical tests used? Choose an item.

**Quality of Written English**

Please indicate the quality of language in the manuscript: Choose an item.

**Declaration of Competing Interests**

Please complete a declaration of competing interests, considering the following questions:

- Have you in the past five years received reimbursements, fees, funding, or salary from an organisation that may in any way gain or lose financially from the publication of this manuscript, either now or in the future?
- Do you hold any stocks or shares in an organisation that may in any way gain or lose financially from the publication of this manuscript, either now or in the future?
- Do you hold or are you currently applying for any patents relating to the content of the manuscript?
- Have you received reimbursements, fees, funding, or salary from an organization that holds or has applied for patents relating to the content of the manuscript?
- Do you have any other financial competing interests?
- Do you have any non-financial competing interests in relation to this paper?

If you can answer no to all of the above, write 'I declare that I have no competing interests' below. If your reply is yes to any, please give details below.

no to all.

I declare that I have no competing interests

I agree to the open peer review policy of the journal. I understand that my name will be included on my report to the authors and, if the manuscript is accepted for publication, my named report including any attachments I upload will be posted on the website along with the authors' responses. I agree for my report to be made available under an Open Access Creative Commons CC-BY license (http://creativecommons.org/licenses/by/4.0/). I understand that any comments which I do not wish to be included in my named report can be included as confidential comments to the editors, which will not be published.

I agree to the open peer review policy of the journal

To further support our reviewers, we have joined with Publons, where you can gain additional credit to further highlight your hard work (see: https://publons.com/journal/530/gigascience). On publication of this paper, your review will be automatically added to Publons, you can then choose whether or not to claim your Publons credit. I understand this statement.

Yes Choose an item.
